# Diagnosis and management of infertility: NICE-adapted guidelines from the Italian Society of Human Reproduction

**DOI:** 10.1186/s12958-023-01179-2

**Published:** 2024-01-05

**Authors:** Stefano Palomba, Paola Viganò, Sandrine Chamayou, Zaira Donarelli, Maria Paola Costantini, Roberto Marci, Paola Piomboni, Egidio Fino, Luigi Montano, Antonino Guglielmino, Edgardo Somigliana, and the SIRU Working Group on Italian Guideline for Infertility, Nicola Arrighi, Anna Biallo, Luca Boeri, Antonella Cinotti, Elisabetta Coccia, Giacomo D’Amico, Cinzia Di Matteo, Vincenzo Favilla, Guglielmino Antonino, Maria Giulia Minasi, Veronica Ricciuto, Marco Sbracia, Francesca Spinella, Maria Vitale, Giulia Eleonora Carmela Zinno

**Affiliations:** 1 Department of Medical-Surgical Science and Translational Medicine, Sapienza University of Rome, Sant’Andrea Hospital, Rome, Italy; 2 Infertility Unit, Fondazione IRCCS Ca’ Granda, Ospedale Maggiore Policlinico, Milan, Italy; 3 HERA Center, Unit of Reproductive Medicine (U.M.R.), Sant’Agata Li Battiati, Catania, Italy; 4 Psychological Counselling Service, University of Palermo, Palermo, and Clinical Psychology Unit, ANDROS Day Surgery Clinic, Palermo, Italy; 5 Italian Society of Human Reproduction, SIRU, Rome, Italy; 6 Department of Translational Medicine, University of Ferrara, Ferrara, Italy; 7 Department of Molecular and Developmental Medicine, University of Siena, Siena, Italy; 8 Unit of Medically Assisted Reproduction, Siena University Hospital, Siena, Italy; 9 Unit and Service of Lifestyle Medicine in Uro-Andrology, Local Health Authority of Salerno, Salerno, Italy; 10 Department of Clinical Sciences and Community Heath, University of Milan, Milan, Italy; 11 Unit of Gynecology, Sant’Andrea Hospital, Via di Grottarossa, 1039 Rome, Italy

**Keywords:** ARTs, Guidelines, Infertility, IVF, Management, Sterility

## Abstract

In Italy the fertility rate is very low, and an increasing number of patients are infertile and require treatments. The Italian Law concerning the safety of patient care, and the professional liability of health professionals, indicates that health professionals must comply with the recommendations set out in the guidelines developed by public and private bodies and institutions, as well as scientific societies and technical-scientific associations of the health professions, except for specific cases. Unfortunately, no guideline for the diagnosis and the management of infertility is currently available in Italy. In 2019, the Italian Society of Human Reproduction pointed out the need to produce Italian guidelines and subsequently approved the establishment of a multidisciplinary and multiprofessional working group (MMWG) to develop such a guideline. The MMWG was representative of 5 scientific societies, one national federation of professional orders, 3 citizens' and patients' associations, 5 professions (including lawyer, biologist, doctor, midwife, and psychologist), and 3 medical specialties (including medical genetics, obstetrics and gynecology, and urology). The MMWG chose to adapt a high-quality guideline to the Italian context instead of developing one from scratch. Using the Italian version of the Appraisal of Guidelines for Research and Evaluation II scoring system, the National Institute of Clinical Excellence guidelines were selected and adapted to the Italian context. The document was improved upon by incorporating comments and suggestions where needed. This study presents the process of adaptation and discusses the pros and cons of the often-neglected choice of adapting rather than developing new guidelines.

**Supplementary Information:**

The online version contains supplementary material available at 10.1186/s12958-023-01179-2.

## Introduction

The Italian Law (Law n. 24, 2017, 8^th^ March) concerning the safety of care and of the patient, and the professional liability of health professionals indicates that health professionals must comply, except for specific cases, with the recommendations set out in the guidelines developed by public and private bodies and institutions as well as scientific societies and technical-scientific associations of the health professions. Regardless of the stakeholders involved in their development, these guidelines must be approved by the National Center for the Clinical Excellence, Quality and Safety of the Care (CNEC, *Centro Nazionale per l’Eccellenza Clinica, la Qualità e la Sicurezza delle Cure*), an institutional organism directly referring to the Ministry of Health. In 2017, this national organism published a methodological manual for guidelines development, a document that has recently been updated [1]. This process is complex and lengthy. To date, after more than 6 years, only 88 guidelines across the entire area of medicine have been approved (https://www.iss.it/linee-guida1).

In Italy, the fertility rate is very low, and an increasing number of patients are infertile and require treatments (https://www.salute.gov.it/imgs/C_17_pubblicazioni_2823_allegato.pdf). Due to the high incidence of infertility, mismanagement of this condition may have significant economic and epidemiological implications. Thus, the development and implementation of guidelines aim to ensure fairness, efficiency, and sustainability. Unfortunately, no CNEC-adopted guideline for the diagnosis and management of infertility is currently available in Italy. Recently, some papers have been published in this field [2, 3]. The first is an expert opinion aimed to summarize the risk factors for infertility and to identify a practical clinical and diagnostic approach for the male and female infertility [2]. The latter is a narrative review aimed to explore the indications, minimum access criteria, and outcomes of the assisted reproductive technologies (ARTs) for the male factor [3].

Based on these considerations, in 2019, the Italian Society of Human Reproduction (SIRU, *Società Italiana di Riproduzione Umana*) decided to submit a document to CNEC with a proposal of guidelines for the diagnosis and management of infertility to be adopted in Italy. This report aims to detail the methodological process followed and the results obtained.

## Methods

Initially, the SIRU steering committee decided to focus on the primary objective of defining Italian guidelines for the diagnosis and management of couple infertility following the provisions of the National System Guidelines System (SNLG, *Sistema Nazionale Linee-Guida*), an activity of CNEC exclusively dedicated to the development of guidelines. All the steps were carried out in accordance with the specific Manual of CNEC, with the latest version being accessible online. (https://www.iss.it/documents/20126/7949265/Manuale+Metodologico+-+marzo+2023.pdf/01f4bc8e-f3e6-66ec-bbe1-e80186908c6c?t=1679921943422)

First, the executive committee of SIRU spread and shared the idea to develop the guidelines among the members of the Society. A special fund was established to collect the necessary resources. This funding was exclusively open to personal contributions, whereas financial resources from commercial companies could not be accepted. In addition, SIRU itself participated to partially cover the expenditures. Overall, a total of €43,950 was collected and used for the process. The fund was mainly employed for a contract with a company specialized in public health activities and guidelines development. SIRU is a young Society, and the executive committee deemed it essential to be supported by a highly professional partner. For this purpose, the Italian Team for the Evidence-Based Medicine (GIMBE, *Gruppo Italiano per la Medicina Basata sulle Evidenze* - https://www.gimbe.org/) was chosen for its undisputed reputation in this field in Italy.

A Multidisciplinary and Multiprofessional Working Group (MMWG) was subsequently defined according to principles of professional and specialist representation, as well as corporate. The MMWG was representative of 5 scientific societies and one national federation of professional orders, 3 citizens' and patients' associations, 5 professions (including lawyer, biologist, doctor, midwife, and psychologist), and 3 medical specialties (including medical genetics, obstetrics and gynecology, and urology).

Each component of the MMWG had to declare any potential conflict of interest before the first MMWG meeting. Having conflicts of interest was not an exclusion criterion from the MMWG, but they had to be transparent to the whole group and were handled in a case-by-case basis, depending on the specific item being evaluated.

The full identity of the MMWG’ members will be made available upon reasonable request from the corresponding author. The MMWG strictly followed the different steps of the process of elaboration of the guideline through subgroups meetings (3 for each geographical macro-areas) followed by discussions during plenary meetings to achieve the consensus. These meetings were initially conducted in person but then shifted to teleconferences after the advent of the COVID-19 outbreak.

Based on the CNEC methodology, the MMWG initially chose to adapt an already available guideline of good methodological quality to the Italian context rather than developing a new independent guideline. Several outstanding guidelines on the same topic were available in other countries, and it was deemed more efficient and sensible to adapt them to the Italian context. It was recognized that drafting new guidelines could be a very time-consuming procedure requiring a significant investment of economic and human resources, which were well beyond those available for the project.

The process of searching and selecting the reference guideline consisted of the following steps: 1. systematic search for available guidelines on infertility management; 2. quality assessment of the identified guidelines; and 3. selection of the best guidelines to use as reference.

To conduct the search, MMWG members screened articles and performed searches using key terms such as "infertility" or "sterility" matched with "guideline" on main websites, including PubMed, Web of Science, Google Scholar, Cochrane Library. All articles that referred to guidelines for the diagnosis and management of infertile couples were screened without language restriction. Additional journal articles were identified from references of the included documents. Full texts of eligible articles were evaluated, and only relevant papers were carefully assessed. The guidelines were then rated for quality using the Italian version [4] of the Appraisal of Guidelines for Research and Evaluation II (AGREE II) scoring system [5, 6]. The guideline with the highest AGREE II score was chosen as the reference document. Subsequently, the MMWG adapted the reference document to the Italian context using the methodology for adapting the recommendations from international guidelines developed by the GIMBE Foundation (https://www.gimbe.org/pagine/569/it/agree-ii). In particular, the MMWG proceeded as follows: 1. deleted recommendations considered not relevant to the guideline aim (and reported the reason for each case ) or inopportune in the country or considered debatable because of lack of valid local epidemiological information; 2. identified new issues potentially relevant for the Italian context and not assessed in the reference guideline; 3. evaluated the applicability of each specific recommendation, reporting any potential obstacle (scientific, structural, technological, organization, professional, regulatory, orographic, socio-cultural, or others) and 4. changed the original recommendations not applicable in Italy and indicated the reasons. All recommendations were analyzed according to the Italian legislation and availability of drugs. The presence of process facilitators was also discussed and analyzed.

New clinical issues could be developed if they were deemed to be uncovered by the selected guideline. The clinical issues were addressed according to the Population, Intervention, Comparison and Outcomes (PICO) model [7]. Specifically, the “Population” comprised of infertile couples, the “Intervention” encompassed each strategy, procedure, or treatment employed to diagnose or treat infertility, the "Comparison" involved neither intervention nor placebo/sham arm or another potentially active intervention, and the “Outcomes” were ranked by importance in evaluating intervention effects case-by-case. Published systematic reviews and meta-analyses were used. No attempts were made to perform new independent systematic reviews.

A new extensive search of systematic reviews, with or without meta-analyses, was performed using the same websites mentioned above to update the referral guideline. Data published up to January 31, 2020, were collected, analyzed, interpreted, and integrated into the referral guideline. The MMWG established that these new questions and the updating process would not pertain to pre-implantation diagnosis of genetic or structural diseases. The complete methodological process followed the “GRADE-ADOLOPMENT approach” based on the Grading of Recommendations Assessment, Development and Evaluation (GRADE) Evidence to Decision (EtD) frameworks [8]. It was developed through plenary and sub-group meetings (3 for each geographical macro-areas). The subdivision of the activity by subgroup concerned only the screening of the systematic reviews identified. The other phases were carried out in a unified group. Any disagreements were resolved by consensus methods at the plenary meetings.

The SIRU guideline was subsequently subjected to a public consultation with the aim of collecting feedback on the preliminary version of the recommendations, as well as to evaluate their applicability and feasibility. This review was carried out through an online platform containing all the clinical recommendations. SIRU invited 241 stakeholders, including 2 bioethicists, 40 biologists, 127 physicians (endocrinologists, gynecologists, oncologists, urologists, immunologists), 6 midwives, 7 psychologists, 15 citizen and patient representatives, and 44 pharmaceutical and biomedical industry representatives. Each participant was asked to provide personal, professional, and contact data, and to list any changes performed to the clinical recommendations, indicating the related reasons, including possible obstacles to their application, and any added reference. The MMWG analyzed the proposed changes and integrated those deemed appropriate in the final recommendations.

After the public consultation, the document was submitted to an external review. The purpose of the external review was to improve the quality of the guidelines and to collect feedback on the draft version of the recommendations. The CNEC identified the Italian Society of Gynecology and Obstetrics (SIGO, *Società Italiana di Ginecologia e Ostetricia*) as main external reviewer. Thus, the document was sent to the SIGO and was required to verify its content having as reference the Methodological Manual of the Guidelines developed by the CNEC. SIGO submitted the document to the members of its own Special Interest Group as well as other representative professional societies, including the Italian Society of Embryology, Reproduction and Research (SIERR, *Società Italiana di Embriologia, Riproduzione e Ricerca*), the Italian Society of Endocrinology (SIE, *Società Italiana di Endocrinologia*), the Italian Society of Andrology and Sexual Medicine (SIAMS, *Società Italiana di Andrologia e Medicina della Sessualità*), and the Luca Coscioni Association. The MMWG replied in a rebuttal letter addressing the suggestions and comments received, including those considered relevant in the final version of the document.

 SIRU decided to update the guidelines every two years after publication, according to the updating of the original guideline. This process should aim to integrate new scientific evidence to support modifying pre-existing recommendations or to draft novel recommendations. The process should also aim to address any inconsistencies that may emerge from subsequent Italian guidelines prepared by other scientific societies or result from new legislation and case law developments.

## Results

After evaluation of the available guidelines, the National Institute for Health and Care Excellence (NICE) guidelines resulted to have the highest AGREE II score and was selected as the guiding reference for this project. In particular, the last update of the NICE guidelines in September 2017 was considered. The full details on the methodological aspects of the NICE guideline are available online on the following websites: www.nice.org.uk/guidance/cg156/evidence/full-guideline-pdf-188539453, www.nice.org.uk/guidance/cg156/evidence/appendices-a-o-pdf-188539454, www.nice.org.uk/guidance/cg156/evidence/appendix-h-pdf-188539455, www.nice.org.uk/guidance/cg156/evidence/appendix-m-pdf-188539456, and www.nice.org.uk/guidance/cg156/evidence/appendix-n-pdf-188539457.

Five original recommendations were deleted because considered unsuitable for inclusion. Specific reasons were as follows: absence of local epidemiological data (n. 3), drug not available (n. 1) and recommendation deemed redundant by the panel (n. 1). They are detailed in Table 1, together with reasons for being discarded.

**Table 1:** The following NICE recommendations have been removed from the SIRU guidelines due to being deemed inappropriate with specific reasons

**NICE CG156 – recommendation number**	**Delated recommendation**	**Reason**
1.3.13.1.	Before undergoing uterine instrumentation, women should be offered screening for Chlamydia trachomatis using an appropriately sensitive technique.	Panel decision (unanimity). Data on Chlamydia trachomatis prevalence are not available in Italy. The adoption of this recommendation in the absence of local data would have contrasted with the local strong plea for limiting the use of antibiotics.
1.3.13.2.	If the result of a test for Chlamydia trachomatis is positive, women and their sexual partners should be referred for appropriate management with treatment and contact tracing.	
1.3.13.3.	Prophylactic antibiotics should be considered before uterine instrumentation if screening has not been carried out.	
1.5.2.9.	The effectiveness of pulsatile gonadotrophin-releasing hormone in women with clomifene citrate-resistant polycystic ovary syndrome is uncertain and is therefore not recommended outside a research context.	Panel decision (unanimity). Drug unavailable in Italy.
1.17.2.4.	Limit drugs used for controlled ovarian stimulation in IVF treatment to the lowest effective dose and duration of use.	Panel decision (unanimity). Deemed redundant, the concept was already included in the NICE recommendation 1.17.1.3. (17.1.3. for SIRU guidelines).

Overall, for the process of adjournment, 829 systematic reviews were identified and screened. A total of 35 items were analyzed and included in the guideline. Specifically, 13 systematic reviews led to the modification of a recommendation [9–21], whereas other 22 systematic reviews were employed to develop new recommendations [22–43]. Other further documents were discussed and cited. They included three guidelines from the European Society of Human Reproduction and Embryology (ESHRE) [44], American Society for Reproductive Medicine (ASRM) [45], and Italian Association of Medical Oncology (AIOM, *Associazione Italiana di Oncologia Medica)* (https://www.iss.it/documents/20126/8403839/LG296_Fertilit%C3%A0_PZ_Oncologici_agg2021.pdf/29e2ba98-a209-8805-cc0b-706f6c7ee2a5?version=1.0&t=1678805156827).

The modified recommendations as well as those newly developed, are presented in Tables 2 and 3, respectively, detailing the choices made in each case and the documents considered. Table 3 also includes studied [46, 47] discussed during the panel discussion and the public consultation (see below).

**Table 2.** NICE recommendations modified and included in the SIRU guidelines with specific reason.

**NICE CG156 – recommendation number**	**SIRU guideline – recommendation number**	**Modified recommendation** ^a^	**Reason**
1.2.5.1.	2.5.1.	People who are concerned about their fertility should be informed that there is no consistent evidence of an association between consumption of caffeinated beverages (tea, coffee and colas) and fertility problems*. However, assumption of more than 2-3 cups of coffee (200-300 mg of caffeine) daily is associated with an increased risk of early pregnancy loss.*	Based on Lyngsø et al., 2017 [9]
3.10.1.	1.3.9.1.	People undergoing IVF treatment must undergo testing for HIV, hepatitis B, hepatitis C *and syphilis.*	Panel decision (unanimity). Based on the recommendations of the Italian Ministry of Health (DLGS 85/2012 e DPR 131/2019).
1.4.2.2.	4.2.2.	*Offer surgery for varicocele to improve the chance of natural conception in men with semen abnormalities, clinically remarkable varicocele, and a partner younger than 35 and a good ovarian reserve.*	Based on Kim et al., 2013 [10]
1.5.2.2.	5.2.2.	Offer women with WHO Group 2 anovulatory infertility 1 of the following treatments, taking into account potential adverse effects, ease and mode of use, the woman's BMI, and monitoring needed: • clomifene citrate or • metformin	Based on Sharpe et al., 2020 [11]
1.5.2.6.	5.2.6.	For women with WHO Group 2 ovulation disorders who are known to be resistant to clomifene citrate, consider one of the following second-line treatments, depending on clinical circumstances and the woman's preference: • laparoscopic ovarian drilling or • combined treatment with clomifene citrate and metformin or • gonadotrophins	Based on Bordewijk et al., 2020 [12]
1.8.1.3.	8.1.3.	Advise women with unexplained infertility who are having regular unprotected sexual intercourse to try to conceive for a total of 2 years (this can include up to one year before their fertility investigations) before IVF will be considered. *Consider a shorter period for women older than 35.*	Panel decision (no unanimity). Discrimination of women aged 40-42 was deemed unfair, complicated and in contrast with the national plea for facilitating motherhood to counteract the falling birth rate.
1.8.1.4.	8.1.4.	Offer IVF treatment to women with unexplained infertility who have not conceived after 2 years (this can include up to 1 year before their fertility investigations) of regular unprotected sexual intercourse. *Consider a shorter period for women older than 35.*	Panel decision (no unanimity). Discrimination of women aged 40-42 was deemed unfair, complicated and in contrast with the national plea for facilitating motherhood to counteract the falling birth rate.
1.9.1.3.	9.1.3.	For people with unexplained infertility, mild endometriosis or mild male factor infertility, who are having regular unprotected sexual intercourse: • do not routinely offer intrauterine insemination, either with or without ovarian stimulation (exceptional circumstances include, for example, when people have social, cultural or religious objections to IVF) • advise them to try to conceive for a total of 2 years (this can include up to 1 year before their fertility investigations) before IVF will be considered. *Consider a shorter period for women older than 35.*	Panel decision (no unanimity). Discrimination of women aged 40-42 was deemed unfair, complicated and in contrast with the national plea for facilitating motherhood to counteract the falling birth rate.
1.11.1.1.	11.1.1.	When considering IVF as a treatment option for people with fertility problems, discuss the risks and benefits of IVF	Panel decision (unanimity). HEFA code of practice is not used in Italy.
1.11.1.3 and 1.11.1.4.	11.1.3.	In women aged < *43* years offer 3 full cycles of IVF, with or without ICSI. If the woman reaches the age of *43* years during treatment, complete the current full cycle but do not offer further full cycles. In women aged 40 to 42 years IVF, with or without ICSI, provided the following criteria are fulfilled: • there is no evidence of low ovarian reserve • there has been a discussion of the additional implications of IVF and pregnancy at this age.	Panel decision (no unanimity). Discrimination of women aged 40-42 was deemed unfair, complicated and in contrast with the national plea for facilitating motherhood to counteract the falling birth rate.
1.12.3.3.	12.5.5.	When using gonadotrophins for ovarian stimulation in IVF treatment: use an individualised starting dose of follicle-stimulating hormone, based on factors that predict success, such as: - age - BMI - presence of polycystic ovaries - ovarian reserve Do not use a dosage of follicle-stimulating hormone of more than *300* IU/day	Based on Lensen et al., 2018 [13]
1.12.3.7.	12.3.7.	Do not use growth hormone as adjuvant treatment in IVF protocols.	Based on Liu et al., 2018 [14]
1.12.5.2.	12.5.2.	For the safe practice of administering sedative drugs refer to *local Italian Guidelines*	Panel decision (unanimity). No specific reference is reported because these guidelines are under development.
1.12.5.3.	12.5.3.	Women should not be offered follicle flushing because this procedure does not increase the numbers of oocytes retrieved or pregnancy rates, and it increases the duration of oocyte retrieval and associated pain.	Based on Georgiou et al., 2018 [15]
1.12.5.5.	12.5.5.	Assisted hatching *in fresh embryos* is not recommended because it has not been shown to improve pregnancy rates. *Evidence for frozen embryos is yet inconclusive.*	Based on Zeng et al., 2018 [16]
1.12.6.4.	12.6.4.	Evaluate embryo quality, at both cleavage and blastocyst stages, according to ESHRE recommendation	Based on ESHRE Special Interest Group, 2017 [44]
1.12.7.3.	12.7.3.	Inform women undergoing IVF treatment that the evidence does not support continuing any form of treatment for luteal phase support beyond *5* weeks' gestation.	Based on Watters et al., 2020 [17]
1.13.1.1.	13.1.1.	The recognised indications for treatment by intracytoplasmic sperm injection (ICSI) include: severe deficits in semen quality, obstructive azoospermia, non-obstructive azoospermia *and the use of frozen eggs.* In addition, treatment by ICSI should be considered for couples in whom a previous IVF treatment cycle has resulted in failed or very poor fertilisation.	Panel decision (unanimity). Based on Italian legislation and regulations.
1.15.2.1.	15.2.1.	*Oocytes donation programs should adhere to the according to the Italian legislation and regulations (D. Lgs. 16/2010, D. Lgs. 85/2012 and DPR 131/2019)*.	Panel decision (unanimity). Based on Italian legislation and regulations.
1.16.1.1.	16.1.1.	When considering and using cryopreservation for people before starting chemotherapy or radiotherapy, that is likely to affect their fertility, follow recommendations *of the SNLG.*	Based on AIOM guidelines. Available at: https://www.iss.it/documents/20126/8403839/LG296_Fertilit%C3%A0_PZ_Oncologici_agg2021.pdf/29e2ba98-a209-8805-cc0b-706f6c7ee2a5?version=1.0&t=1678805156827 (National guideline on fertility preservation available on the website of the Ministry of Health).
1.16.1.7.	16.1.8.	When using cryopreservation to preserve fertility in people diagnosed with cancer, use sperm or oocytes *or ovarian cortex.*	Panel decision (unanimity). Embryos cannot be stored in a fertility preservation context based on the Italian Legislation (Law n. 40/2004).
1.16.1.12 and 1.16.1.13.	16.1.14	Include in the informed consent the duration of freezing and the modalities to renovate the cryopreservation.	Panel decision (unanimity). Unclear Italian legislation. Risks of litigations if a rigid threshold of duration is given.
1.17.2.3.	17.2.4.	Inform people who are considering IVF treatment that *newborns have a modest increase in the risk of malformations. It has not yet been clarified whether the risk is related to the procedure or whether it is related to the condition of infertility.*	Based on Liang et al., 2017, Hoorsan et al., 2017, Chen et al., 2018, and Giorgione et al., 2018 [18–21]

^a^The changes/modifications are reported in *italics* when added and as text when deleted

**Table 3:** New recommendations included in the SIRU guidelines with specific reason

**SIRU guideline – recommendation number**	**New recommendation**	**Reason**
2.10.1.	Exposure to mercury may interfere with female and male fertility. Attention should be given to professional and dietary exposure to mercury.	Based on Henriques et al., 2019 [22]
2.14.	Inform women who had a previous cesarean section that they should wait for at least 10 months after the intervention prior to initiate pregnancy seeking to reduce the risk of uterine rupture.	Based on Matorras et al., 2019 [23]
3.3.1.	Despite some evidence on the association between HPV infection and female and male infertility, there is no indication to investigate its presence because the infection does not modify the subsequent fertility work-up.	Based on Weinberg et al., 2020 [24] and Yuan et al., 2020 [25]
3.9.3.	Diagnosis of proximal tubal block does not impact on the success of intrauterine insemination. These women should receive the same management as those with bilateral patency. Conversely, the diagnosis of distal block halves the chance of success.	Based on Tan et al., 2019 [26]
4.2.3.	Offer surgery for varicocele to infertile men with an indication to IVF, including men with non-obstructive azoospermia.	Based on Esteves et al., 2016 [27] and Kirby et al., 2016 [28]
10.5.4.	Inform women scheduled for IVF that a regular physical exercise in the period preceding the attempt can increase the success of the procedure.	Based on Rao et al., 2018 [29]
11.1.4.1	In good prognosis women (such as young women who had a prior clinical pregnancy for IVF), consider continuing beyond the limit of three full IVF cycles. This decision should be carefully evaluated and justified taking into utmost consideration the balance between risks and benefits.	Panel decision (unanimity). Recommendation long debated within the panel [46]
12.3.8.	Consider prescribing myo-inositol prior to IVF because it can reduce the total dose of administered gonadotropins.	Based on Zheng et al., 2017 [30] and Laganà et al., 2018 [31]
12.4.2.	Consider trigger with GnRH agonists in women at risk of ovarian hyperstimulation syndrome.	Based on Mizrachi et al., 2020 [32]
12.4.5.	Consider dopamine agonists to prevent ovarian hyperstimulation syndrome in women at risk.	Based on Tang et al., 2016 [33]
12.6.10.	Inform women that single embryo transfer does not fully protect from twin pregnancy. IVF pregnancies are at 2–3-time higher risk of monozygotic twins.	Based on Hviid et al., 2018 [34] and Busnelli et al., 2019 [35]
12.6.11.	Inform women that IVF is associated with a higher risk of placental anomalies (placenta praevia, placental abruption and abnormal cord insertion).	Based on Vermey et al., 2019 [36]
12.6.14.	Inform women on the possibility to perform preimplantation genetic screening for aneuploidies but clarify that it cannot increase the chance of pregnancy.	Decision panel (no unanimity). Based on Lee et al., 2015 [37], and Cornelisse et al., 2020 [38]. Recommendation long debated within the panel [47]
15.3.4.	Women undergoing oocytes donation should be informed about the increased obstetrics risks, including preterm birth, hypertensive disorders and low weight newborns. The counseling should be tailored to the specific condition of the woman.	Based on Jeve et al., 2016 [39], Masoudian et al., 2016 [40] and Mascarenhas et al., 2017 [41]
16.1.4.	Consider the provision of information with written material and audio-visual media because they actively involve and empower the patients and can improve fertility preservation decision-making.	Based on Wang et al., 2019 [42]
16.1.12	Offer ovarian cortex freezing in prepubertal or young girls who are preparing for medical treatment for cancer that is likely to make them infertile if: • they are well enough to undergo surgery • this will not worsen their condition and • enough time is available before the start of their cancer treatment.	Based on ASRM [45] and AIOM guidelines (see before).
17.2.3.	Even if rare, inform people who are considering IVF treatment with or without ICSI that newborns are at increased risk of imprinting disorders.	Based on Cortessis et al., 2018 [43]

Twenty-five recommendations were modified for new evidence available (n. 11, including updated systematic reviews and available guidelines), and after panel decision (n. 14). Specifically, the panel decisions were mainly taken for issues conflicting with Italian legislation and regulations (Table 2). Finally, we introduced 17 new recommendations of which 15 were supported by novel evidence (including updated systematic reviews and available guidelines) and 2 were based on the panel opinion (Table 3).

The public consultation of the SIRU guideline was attended by 128 external reviewers, including 2 bioethicists, 28 biologists, 71 doctors (endocrinologists, gynecologists, oncologists, urologists, immunologists), 4 midwives, 3 psychologists, 8 representatives of citizens and patients and 12 representatives of pharmaceutical and biomedical industries. After revisions following the public consultation, the document underwent external review by 30 experts. The MMWG evaluated and assessed the comments received and prepared a comprehensive response. Comments and suggestions were categorized into 255 major points and a rebuttal letter was prepared in response. Any suggestions that were deemed relevant were included in the final version of the document.

For reference, the definitive specific recommendations are detailed in the Supplementary file, reported in Italian to prevent potential inaccuracies in translation. Tables 1, 2 and 3 in the original NICE document detail all changes made, allowing readers to easily access the final English version of the document. The entire process took more than two years, beginning in May 2019 and concluding in August 2021 with submission to CNEC. Unfortunately, the document was rejected by CNEC one year later. The evaluation covered various quality parameters, including context of the scope, methodology, stakeholders’ involvement, clarity in argumentation, and absence of interest. Applicability received the lowest score, but no specific reasons for this were provided.

## Discussion

Clinical guidelines are thought to improve the quality-of-care patients receive. By summarizing evidence on a specific topic, guidelines make it easier for clinicians to make informed, evidence-based decisions for their patients in a timely manner. Guidelines not only promote proven interventions but also discourage ineffective or potentially harmful ones. Additionally, guidelines can empower patients, influence public policy, improve the consistency of care, and drive the development of disease performance measures and evaluations [48]. Evidence-based guidelines are a crucial tool in assisting physicians, policymakers, and patients in every area of medicine, but they are especially important in the field of reproductive medicine. This is because infertility remains a significant challenge worldwide, as noted by the World Health Organization (https://www.who.int/news-room/fact-sheets/detail/infertility). In many countries, access to and quality of interventions addressing infertility are limited. Unfortunately, the diagnosis and treatment of infertility are not always prioritized, and reproductive health strategies may not be covered by public health systems. Other issues related to infertility include the high cost of drugs and the lack of access to high-tech equipment. In Italy, ART procedures are not yet covered by the National Public Health system, although some regions do provide support. The availability of ART within the public health system varies greatly throughout the country, as local regions may choose to offer procedures if they are deemed sustainable by local governments. Despite an equitable distribution of funds to all regions, some provide full and unlimited support for ART (such as Lombardy), while others offer no support at all (such as Sicily). However, there are several private fertility centers throughout the country that offer high-quality care in fertility treatments. Thus, to mitigate inequities and disparities in access to fertility care, SIRU has developed guidelines with the patient as the primary focus. Two reasons prompted SIRU to establish these guidelines: (i) the implementation of Italian Law No. 24 in 2017, which regulated the development of guidelines by scientific societies, and the establishment of a system dedicated to their assessment and implementation (the CNEC); and (ii) the lack of clarity surrounding ART procedures due to the rules and limitations of Italian Law No. 40/2004, which governs medically assisted reproduction. The law has always been a focus of legal issues, particularly related to banned procedures. The positions of the Constitutional Court have mostly overturned the procedures forbidden by the Law, but pragmatical "grey" areas remain. Guidelines were developed to provide appropriate guidance on these aspects. Based on these factors, and considering the limited financial resources available, the panel of experts and representatives who composed the MMWG decided that adapting existing guidelines to the national context was the most appropriate approach. It was recognized that guidelines often take a significant amount of time to develop, and this decision was viewed as the most practical and efficient solution.

This paper provides a detailed account of the process involved in adapting the NICE guidelines entitled "Fertility: assessment and treatment for people with fertility problems" to the Italian context. Although less demanding than developing a new guideline, the process was lengthy and required significant resources, which were unfortunately further prolonged by the COVID-19 outbreak, leading to intense debates among the involved parties. The process involved up to 200 individuals and resulted in the deletion of five recommendations, update of 25 recommendations, and development of 17 new recommendations. The final document consisted of 217 recommendations, mostly overlapping with the original NICE guidelines but updated and adapted to the Italian context.

Unfortunately, to date, the guideline implementation has not been possible, and we have yet to assess its applicability and overall impact. In May 2022, the CNEC provided a negative evaluation of the document, without providing the opportunity for revision, which is not in compliance with the normal process outlined in their manual. The main concern raised was the limited applicability of the document. The decision was surprising since the MMWG had provided an adaptation of a guideline used in UK for several years (with the last version dating back to 2013) with no recent adjustments required. In addition, the changes made by the MMWG to the NICE guidelines affected only a minority of recommendations and were not critical. On the other hand, the negative assessment of CNEC in relation to SIRU guidelines has faced criticism at the societal level for the decision to adapt guidelines instead of developing new ones. Choosing the NICE guidelines as the reference document was likely not the problem. In other words, the negative evaluation may not have been dependent on which guidelines the MMWG had chosen to use. A recent review published in *Heart* [49] described the principles and processes of clinical guideline development at NICE. Notably, NICE recommendations consider both the clinical effectiveness and the cost-effectiveness of interventions [49]. The guidelines are developed by an independent panel of healthcare professionals supported by a team that includes project managers, information specialists, systematic reviewers, and health economists. Moreover, NICE selects a limited number of high-impact questions to answer through the review of evidence, rather than exhaustively covering a particular topic. These factors alone can ensure the quality of the development process [49].

On the other hand, we must acknowledge that the decision made by CNEC was in response to challenges posed by the Italian healthcare system. ART procedures are only covered at certain infertility centers in specific regions, making it difficult to apply the SIRU guidelines across the entire country. Therefore, it is understandable that CNEC had to adapt the guidelines to fit the unique Italian context. Since broad applicability is crucial for CNEC-endorsed guidelines, their decision is partly understandable. While SIRU guidelines may be useful for regions covering ART, they may not be relevant for regions that do not. However, it is worth noting that the government recently recognized ART as a supported treatment, and all regions are now mandated to provide it to citizens starting from January 1st, 2024. With this in mind, we remain optimistic that the evaluation of SIRU guidelines' limited applicability will be reassessed in due course.

Another possible explanation for CNEC’s decision could be related to the significant legal disparities in how infertility issues are regulated in Italy and the UK. While Court rulings may have addressed some of the limitations of Italian Law 40, it remains in effect. The CNEC may have viewed this aspect as a strong barrier to the applicability of the NICE guidelines as the legislation regulating ART in the UK does not ban procedures that are prohibited under the Italian law.

Regardless of the reasons behind the rejection of the SIRU guidelines, we have chosen to publish our efforts as a model for others. Additionally, we advocate for greater use of the adaptive approach to guidelines in the future, as this approach permits the use of updated, tailored, and high-quality guidelines without significant economic or human resource investments. Adopting the adaptive approach to guidelines could be a highly appealing option for low- and medium-income countries. However, a careful evaluation of the pros and cons of adopting versus developing novel guidelines should be conducted before embarking on a challenging endeavor, such as the one presented herein. Infertility care may be particularly critical in this regard, given the disparities in accesses, governmental policy interest, and legislation on this topic across different countries. Finally, since no official guidelines for infertility currently exist in Italy, these adapted guidelines – based on the NICE ones – will serve as the formal clinical guidelines of SIRU until new guidelines are released.

### Supplementary Information

**Additional file 1: Supplementary document.** Linee guida per la diagnosi e il trattamento dell’infertilità. NICE-adapted guidelines of the SIRU in Italian version.

## Data Availability

The datasets used and/or analyzed during the current study are available from the corresponding author on reasonable request.

## Abbreviations

AGREE II: Appraisal of Guidelines for Research and Evaluation II
AIOM: Italian Association of Medical Oncology
ARTs: assisted reproductive technologies
ASRM: American Society of Reproductive Medicine
CNEC: National Center for the Clinical Excellence, Quality and Safety of the Care
EtD: Evidence to Decision
GIMBE: Italian Team for the Evidence-Based Medicine
GRADE: Grading of Recommendations Assessment, Development and Evaluation
ICSI: intracytoplasmic sperm injection
IVF: in vitro fertilization
MMWG: Multidisciplinary and Multiprofessional Working Group
NICE: National Institute of Clinical Excellence
PDTA: Diagnostic-Therapeutic-Care Pathways
PICO: Population, Intervention, Comparison and Outcomes
SIRU: Italian Society of Human Reproduction
SNLG: National System Guidelines System

## References

[1] Coclite D, Napoletano A, Fauci AJ, Graziano G, Iannone P. Manuale metodologico per la produzione di linee guida di pratica clinica. 2017. Adjourned form available at: https://www.iss.it/documents/20126/7949265/Manuale+Metodologico+-+marzo+2023.pdf/01f4bc8e-f3e6-66ec-bbe1-e80186908c6c?t=1679921943422.

[2] Garolla A, Pizzol D, Carosso AR, Borini A, Ubaldi FM, Calogero AE, Ferlin A, Lanzone A, Tomei F, Engl B, Rienzi L, De Santis L, Coticchio G, Smith L, Cannarella R, Anastasi A, Menegazzo M, Stuppia L, Corsini C, Foresta C (2021). Practical clinical and diagnostic pathway for the investigation of the infertile couple. Front Endocrinol (Lausanne).

[3] Mazzilli R, Rucci C, Vaiarelli A, Cimadomo D, Ubaldi FM, Foresta C, Ferlin A (2023). Male factor infertility and assisted reproductive technologies: indications, minimum access criteria and outcomes. J Endocrinol Invest.

[4] Cartabellotta A (2011). AGREE II: come valutare la qualità delle linee-guida [AGREE II: assessing the quality of practice guidelines]. Recenti Prog Med.

[5] Brouwers MC, Kho ME, Browman GP, Burgers JS, Cluzeau F, Feder G, Fervers B, Graham I, Grimshaw J, Hanna SE (2012). The global rating scale complements the AGREE II in advancing the quality of practice guidelines. J Clin Epidemiol.

[6] Brouwers MC, Kerkvliet K, Spithoff K (2016). The AGREE Reporting Checklist: a tool to improve reporting of clinical practice guidelines. BMJ.

[7] Frandsen TF, Bruun Nielsen MF, Lindhardt CL, Eriksen MB (2020). Using the full PICO model as a search tool for systematic reviews resulted in lower recall for some PICO elements. J Clin Epidemiol.

[8] Schünemann HJ, Wiercioch W, Brozek J, Etxeandia-Ikobaltzeta I, Mustafa RA, Manja V, Brignardello-Petersen R, Neumann I, Falavigna M, Alhazzani W, Santesso N, Zhang Y, Meerpohl JJ, Morgan RL, Rochwerg B, Darzi A, Rojas MX, Carrasco-Labra A, Adi Y, AlRayees Z, Riva J, Bollig C, Moore A, Yepes-Nuñez JJ, Cuello C, Waziry R, Akl EA (2017). GRADE Evidence to Decision (EtD) frameworks for adoption, adaptation, and de novo development of trustworthy recommendations: GRADE-ADOLOPMENT. J Clin Epidemiol.

[9] Lyngsø J, Ramlau-Hansen CH, Bay B, Ingerslev HJ, Hulman A, Kesmodel US (2017). Association between coffee or caffeine consumption and fecundity and fertility: a systematic review and dose-response meta-analysis. Clin Epidemiol.

[10] Kim KH, Lee JY, Kang DH, Lee H, Seo JT, Cho KS (2013). Impact of surgical varicocele repair on pregnancy rate in subfertile men with clinical varicocele and impaired semen quality: a meta-analysis of randomized clinical trials. Korean J Urol.

[11] Sharpe A, Morley LC, Tang T, Norman RJ, Balen AH (2019). Metformin for ovulation induction (excluding gonadotrophins) in women with polycystic ovary syndrome. Cochrane Database Syst Rev.

[12] Bordewijk EM, Ng KYB, Rakic L, Mol BWJ, Brown J, Crawford TJ, van Wely M (2020). Laparoscopic ovarian drilling for ovulation induction in women with anovulatory polycystic ovary syndrome. Cochrane Database Syst Rev..

[13] Lensen SF, Wilkinson J, Leijdekkers JA, La Marca A, Mol BWJ, Marjoribanks J, Torrance H, Broekmans FJ (2018). Individualised gonadotropin dose selection using markers of ovarian reserve for women undergoing in vitro fertilisation plus intracytoplasmic sperm injection (IVF/ICSI). Cochrane Database Syst Rev..

[14] Liu Y, Hu L, Fan L, Wang F (2018). Efficacy of dehydroepiandrosterone (DHEA) supplementation for in vitro fertilization and embryo transfer cycles: a systematic review and meta-analysis. Gynecol Endocrinol.

[15] Georgiou EX, Melo P, Brown J, Granne IE (2018). Follicular flushing during oocyte retrieval in assisted reproductive techniques. Cochrane Database Syst Rev..

[16] Zeng M, Su S, Li L (2018). The effect of laser-assisted hatching on pregnancy outcomes of cryopreserved-thawed embryo transfer: a meta-analysis of randomized controlled trials. Lasers Med Sci.

[17] Watters M, Noble M, Child T, Nelson S (2020). Short versus extended progesterone supplementation for luteal phase support in fresh IVF cycles: a systematic review and meta-analysis. Reprod Biomed Online.

[18] Liang Y, Chen L, Yu H, Wang H, Li Q, Yu R, Qin J (2017). Which type of congenital malformations is significantly increased in singleton pregnancies following after in vitro fertilization/intracytoplasmic sperm injection: a systematic review and meta-analysis. Oncotarget.

[19] Hoorsan H, Mirmiran P, Chaichian S, Moradi Y, Hoorsan R, Jesmi F (2017). Congenital malformations in infants of mothers undergoing assisted reproductive technologies: a systematic review and meta-analysis study. J Prev Med Public Health.

[20] Chen L, Yang T, Zheng Z, Yu H, Wang H, Qin J (2018). Birth prevalence of congenital malformations in singleton pregnancies resulting from in vitro fertilization/intracytoplasmic sperm injection worldwide: a systematic review and meta-analysis. Arch Gynecol Obstet.

[21] Giorgione V, Parazzini F, Fesslova V, Cipriani S, Candiani M, Inversetti A, Sigismondi C, Tiberio F, Cavoretto P (2018). Congenital heart defects in IVF/ICSI pregnancy: systematic review and meta-analysis. Ultrasound Obstet Gynecol..

[22] Henriques MC, Loureiro S, Fardilha M, Herdeiro MT (2019). Exposure to mercury and human reproductive health: a systematic review. Reprod Toxicol.

[23] Matorras R, Berreteaga L, Laínz L, Exposito A, Martínez L (2019). Influence of Caesarean section-pregnancy interval on uterine rupture risk and IVF pregnancy rates: systematic review and mathematical modelling. Reprod Biomed Online.

[24] Weinberg M, Sar-Shalom Nahshon C, Feferkorn I, Bornstein J (2020). Evaluation of human papilloma virus in semen as a risk factor for low sperm quality and poor in vitro fertilization outcomes: a systematic review and meta-analysis. Fertil Steril.

[25] Yuan S, Qiu Y, Xu Y, Wang H (2020). Human papillomavirus infection and female infertility: a systematic review and meta-analysis. Reprod Biomed Online.

[26] Tang H, Mourad S, Zhai SD, Hart RJ (2016). Dopamine agonists for preventing ovarian hyperstimulation syndrome. Cochrane Database Syst Rev..

[27] Esteves SC, Miyaoka R, Roque M, Agarwal A (2016). Outcome of varicocele repair in men with nonobstructive azoospermia: systematic review and meta-analysis. Asian J Androl.

[28] Kirby EW, Wiener LE, Rajanahally S, Crowell K, Coward RM (2016). Undergoing varicocele repair before assisted reproduction improves pregnancy rate and live birth rate in azoospermic and oligospermic men with a varicocele: a systematic review and meta-analysis. Fertil Steril.

[29] Rao M, Zeng Z, Tang L (2018). Maternal physical activity before IVF/ICSI cycles improves clinical pregnancy rate and live birth rate: a systematic review and meta-analysis. Reprod Biol Endocrinol.

[30] Zheng X, Lin D, Zhang Y, Lin Y, Song J, Li S, Sun Y (2017). Inositol supplement improves clinical pregnancy rate in infertile women undergoing ovulation induction for ICSI or IVF-ET. Medicine (Baltimore).

[31] Laganà AS, Vitagliano A, Noventa M, Ambrosini G, D'Anna R (2018). Myo-inositol supplementation reduces the amount of gonadotropins and length of ovarian stimulation in women undergoing IVF: a systematic review and meta-analysis of randomized controlled trials. Arch Gynecol Obstet.

[32] Mizrachi Y, Horowitz E, Farhi J, Raziel A, Weissman A (2020). Ovarian stimulation for freeze-all IVF cycles: a systematic review. Hum Reprod Update.

[33] Tan J, Tannus S, Taskin O, Kan A, Albert AY, Bedaiwy MA (2019). The effect of unilateral tubal block diagnosed by hysterosalpingogram on clinical pregnancy rate in intrauterine insemination cycles: systematic review and meta-analysis. BJOG.

[34] Hviid KVR, Malchau SS, Pinborg A, Nielsen HS (2018). Determinants of monozygotic twinning in ART: a systematic review and a meta-analysis. Hum Reprod Update.

[35] Busnelli A, Dallagiovanna C, Reschini M, Paffoni A, Fedele L, Somigliana E (2019). Risk factors for monozygotic twinning after in vitro fertilization: a systematicreview and meta-analysis. Fertil Steril.

[36] Vermey BG, Buchanan A, Chambers GM, Kolibianakis EM, Bosdou J, Chapman MG, Venetis CA (2019). Are singleton pregnancies after assisted reproduction technology (ART) associated with a higher risk of placental anomalies compared with non-ART singleton pregnancies? A systematic review and meta-analysis. BJOG.

[37] Lee E, Illingworth P, Wilton L, Chambers GM (2015). The clinical effectiveness of preimplantation genetic diagnosis for aneuploidy in all 24 chromosomes (PGD-A): systematic review. Hum Reprod.

[38] Cornelisse S, Zagers M, Kostova E, Fleischer K, van Wely M, Mastenbroek S (2020). Preimplantation genetic testing for aneuploidies (abnormal number of chromosomes) in in vitro fertilisation. Cochrane Database Syst Rev..

[39] Jeve YB, Potdar N, Opoku A, Khare M (2016). Donor oocyte conception and pregnancy complications: a systematic review and meta-analysis. BJOG.

[40] Masoudian P, Nasr A, de Nanassy J, Fung-Kee-Fung K, Bainbridge SA, El Demellawy D (2016). Oocyte donation pregnancies and the risk of preeclampsia or gestational hypertension: a systematic review and metaanalysis. Am J Obstet Gynecol.

[41] Mascarenhas M, Sunkara SK, Antonisamy B, Kamath MS (2017). Higher risk of preterm birth and low birth weight following oocyte donation: a systematic review and meta-analysis. Eur J Obstet Gynecol Reprod Biol.

[42] Wang Y, Anazodo A, Logan S (2019). Systematic review of fertility preservation patient decision aids for cancer patients. Psychooncology.

[43] Cortessis VK, Azadian M, Buxbaum J, Sanogo F, Song AY, Sriprasert I, Wei PC, Yu J, Chung K, Siegmund KD (2018). Comprehensive meta-analysis reveals association between multiple imprinting disorders and conception by assisted reproductive technology. J Assist Reprod Genet.

[44] ESHRE Special Interest Group of Embryology and Alpha Scientists in Reproductive Medicine. The Vienna consensus: report of an expert meeting on the development of ART laboratory performance indicators. Reprod Biomed Online. 2017;35:494-510. 10.1016/j.rbmo.2017.06.015 28784335

[45] Practice Committee of the American Society for Reproductive Medicine (2019). Fertility preservation in patients undergoing gonadotoxic therapy or gonadectomy: a committee opinion. Fertil Steril..

[46] Cameron NJ, Bhattacharya S, Bhattacharya S, McLernon DJ (2017). Cumulative live birth rates following miscarriage in an initial complete cycle of IVF: a retrospective cohort study of 112 549 women. Hum Reprod.

[47] Munné S, Kaplan B, Frattarelli JL, Child T, Nakhuda G, Shamma FN, Silverberg K, Kalista T, Handyside AH, Katz-Jaffe M, Wells D, Gordon T, Stock-Myer S, Willman S, STAR Study Group (2019). Preimplantation genetic testing for aneuploidy versus morphology as selection criteria for single frozen-thawed embryo transfer in good-prognosis patients: a multicenter randomized clinical trial. Fertil Steril..

[48] Guerra-Farfan E, Garcia-Sanchez Y, Jornet-Gibert M, Nuñez JH, Balaguer-Castro M, Madden K (2023). Clinical practice guidelines: The good, the bad, and the ugly. Injury.

[49] Garbi M (2021). National Institute for Health and Care Excellence clinical guidelines development principles and processes. Heart.

