# Supplementary material for: Diagnosis and management of infertility: NICE-adapted guidelines from the Italian Society of Human Reproduction
Source: Reprod Biol Endocrinol. 2024 Jan 5;22:9. doi: 10.1186/s12958-023-01179-2 (PMC10768082; doi:10.1186/s12958-023-01179-2)
Supplement: Supplementary file 1 — Additional file 1: Supplementary document. Linee guida per la diagnosi e il trattamento dell’infertilità. NICE-adapted guidelines of the SIRU in Italian version. [file 12958_2023_1179_MOESM1_ESM.doc]

| **Linee guida per la diagnosi e il trattamento dell’infertilità** |
| --- |

# RACCOMANDAZIONI CLINICHE

## 1. Princìpi di cura

**1.1. Fornire informazioni**

1.1.1. Fornire le consulenze per problemi di fertilità a entrambi i partner perché le decisioni diagnostiche e terapeutiche riguardano la coppia.

1.1.2. Dare alle persone l’opportunità di prendere decisioni in merito all’assistenza e ai trattamenti attraverso l’accesso a informazioni basate sulle evidenze e sulla legislazione in materia. L’informazione deve essere considerata parte integrante del processo decisionale e avvalersi di documenti scritti o in formato audiovisivo.

1.1.3. Fornire le informazioni sulle opzioni terapeutico-assistenziali in una forma accessibile anche a persone con necessità particolari come, ad esempio, soggetti con disabilità fisiche o sensoriali o con ridotte capacità cognitive o linguistiche.

**1.2. Effetti psicologici dei problemi di fertilità**

1.2.1. Nelle coppie con problemi di fertilità, informare entrambi i partner che lo stress dell’uomo e/o della donna può influenzare la relazione di coppia e, riducendo il desiderio sessuale e la frequenza dei rapporti sessuali, può anche contribuire ai problemi di fertilità.

1.2.2. Informare le persone con problemi di fertilità che può essere di aiuto rivolgersi ad associazioni di pazienti e/o a partecipare a gruppi di sostegno specifici.

1.2.3. Proporre alle persone con problemi di fertilità una consulenza psicologica poiché tali problemi, le indagini diagnostiche e i trattamenti potrebbero causare stress psicologico.

1.2.4. Proporre la consulenza psicologica prima, durante e dopo il processo diagnostico e terapeutico indipendentemente dai risultati di queste procedure.

1.2.5. La consulenza psicologica dovrebbe essere erogata da uno psicologo che non abbia un’influenza diretta sulla gestione clinica assistenziale della persona e/o della coppia con problemi di fertilità.

**1.3. Assistenza primaria e specialistica**

1.3.1. Le persone con problemi di fertilità dovrebbero essere prese in carico da un team multiprofessionale e multidisciplinare per migliorare l’efficacia e l’efficienza del trattamento nonché il grado di soddisfazione.

## 2. Consulenza iniziale alle coppie preoccupate per un ritardo nel concepimento

**2.1. Probabilità di concepimento**

2.1.1. Informare le persone preoccupate per la loro fertilità che oltre l’80% delle coppie nella popolazione generale riesce a concepire entro un anno se:

- La donna ha meno di 40 anni.
- Non utilizzano metodi contraccettivi e hanno rapporti sessuali regolari (ogni 2 o 3 giorni).

Circa la metà delle coppie che non concepisce nel primo anno lo fa nel secondo anno (tasso di gravidanza cumulativo superiore al 90%).

2.1.2. Informare le persone che si stanno sottoponendo a cicli di inseminazione intrauterina (IUI) secondo le indicazioni riportate alla raccomandazione 9.1.1 (problematiche sessuologiche, infettivologiche o donazione di seme) che:

- più del 50% delle donne di età <40 anni concepiscono entro 6 cicli di IUI.
- di quelle che non concepiscono dopo 6 cicli di IUI, circa il 50% lo fa entro ulteriori 6 cicli (tasso di gravidanza cumulativo superiore al 75%).

2.1.3. Informare le persone che si stanno sottoponendo a cicli di IUI che l’utilizzo di liquido seminale fresco è associato a tassi di concepimento più alti rispetto all’utilizzo di liquido seminale crioconservato. Inoltre, informarle che la IUI è associata a tassi di concepimento maggiori rispetto all’inseminazione intracervicale.

2.1.4. Informare le persone preoccupate per la loro fertilità che la fertilità femminile si riduce con l’avanzare dell’età, in particolare dopo i 35 anni. Anche la fertilità maschile si riduce con l’età, seppur in misura minore.

2.1.5. Spiegare e discutere le probabilità di concepimento con le persone preoccupate per la loro fertilità utilizzando la tabella 1 per le coppie che hanno rapporti sessuali e la tabella 2 per quelle che ricorrono alle IUI per le indicazioni riportate alla raccomandazione 9.1.1 (problematiche sessuologiche, infettivologiche o donazione di seme).

| **Età della donna (anni)** | **Gravidanza entro un anno** | **Gravidanza entro due anni** |
| --- | --- | --- |
| 19–26 | 92% | 98% |
| 27–29 | 87% | 95% |
| 30–34 | 86% | 94% |
| 35–39 | 82% | 90% |

***Tabella 1.*** *Tassi cumulativi di gravidanza in funzione del numero di cicli mestruali (un anno è equivalente a 12 cicli) (Dunson et al., 2004)*^1^**[[1]](#footnote-2)**

| **Età della donna (anni)** | **IUI con seme congelato** | |
| --- | --- | --- |
|  | **6 cicli** | **12 cicli** |
| <35 | 63% | 86% |
| 35–39 | 50% | 75% |

***Tabella 2.*** *Tassi cumulativi di gravidanza in funzione del numero di inseminazioni con seme congelato (dati HFEA - https://www.hfea.gov.uk/)*

**2.2. Frequenza e tempistica dei rapporti sessuali o dell’inseminazione intrauterina (IUI)**

2.2.1. Informare le persone preoccupate per la loro fertilità che avere rapporti sessuali vaginali completi ogni 2 o 3 giorni ottimizza le probabilità di concepire.

2.2.2. Per le persone che si stanno sottoponendo a inseminazione intrauterina, l’inseminazione deve essere programmata nel periodo dell’ovulazione.

**2.3. Alcol**

2.3.1. Raccomandare alle donne alla ricerca di una gravidanza di non bere più di 1 o 2 unità[[2]](#footnote-3) di alcol 1 o 2 volte/settimana. Informarle inoltre che gli episodi di intossicazione alcolica in gravidanza possono danneggiare il feto.

2.3.2. Informare gli uomini che un consumo di alcol inferiore a 3 o 4 unità/die non dovrebbe avere effetti importanti sulla qualità del liquido seminale.

2.3.3. Informare gli uomini che un eccessivo consumo di alcol danneggia la qualità del liquido seminale.

**2.4. Fumo**

2.4.1. Informare le donne che fumare riduce la fertilità.

2.4.2. Indirizzare le donne fumatrici a servizi o centri anti-fumo.

2.4.3. Informare le donne che il fumo passivo influisce sulle probabilità di concepire.

2.4.4. Informare gli uomini fumatori dell’associazione tra fumo e riduzione della qualità del liquido seminale, e che smettere di fumare migliorerà il loro stato di salute generale.

**2.5. Assunzione di caffeina**

2.5.1. Informare le persone preoccupate per la loro fertilità che non esistono robuste evidenze di associazione tra consumo di bevande contenenti caffeina (caffè, tè, cola) e problemi di fertilità. L’assunzione di più di 2-3 tazze di caffè (200-300 mg di caffeina) al giorno sembra tuttavia associarsi ad aumentato rischio di aborto spontaneo. **[Aggiornata 2020]**[[3]](#footnote-4)

**2.6. Obesità**

2.6.1. Informare le donne con BMI (indice di massa corporea) ≥30 che probabilmente impiegheranno più tempo per concepire e che si espongono ad aumentati rischi ostetrici.

2.6.2. Informare le donne con BMI ≥30 e anovulazione che il dimagrimento aumenta le probabilità di concepimento.

2.6.3. Informare le donne che partecipare a programmi che prevedano sia consigli nutrizionali che attività fisica moderata aumenta le probabilità di gravidanza rispetto al solo dimagrimento.

2.6.4. Informare gli uomini con BMI ≥30 che potrebbero avere problemi di fertilità.

**2.7. Basso peso corporeo**

2.7.1. Informare le donne con BMI <19 e che hanno cicli mestruali irregolari/assenti che un incremento del peso corporeo aumenta le probabilità di concepimento. In presenza di franchi disturbi del comportamento alimentare, considerare un approccio multidisciplinare.

**2.8. Biancheria intima aderente**

2.8.1. Informare gli uomini che un’elevata temperatura scrotale si associa ad una ridotta qualità del liquido seminale. Tuttavia, non ci sono chiare evidenze che indossare biancheria intima non aderente migliori la fertilità.

**2.9. Attività professionale**

2.9.1. Alcuni lavori comportano esposizioni a condizioni di rischio che possono ridurre la fertilità maschile o femminile. Occorre indagare l’attività professionale delle persone preoccupate per la loro fertilità e fornire loro le opportune indicazioni.

**2.10. Esposizione al mercurio**

2.10.1. Il mercurio e gli inquinanti associati possono interferire con la fertilità maschile e femminile. Particolare rilievo deve essere dato alle esposizioni al mercurio sia in termini di attività professionale che di abitudini alimentari. **[Nuova 2020]**[[4]](#footnote-5)

**2.11. Utilizzo di farmaci prescritti, da banco e di droghe ricreative**

2.11.1. L’uso di diversi farmaci e sostanze stupefacenti interferisce con la fertilità maschile e femminile. Chiedere alle persone preoccupate per la loro fertilità se ne fanno uso e, in caso affermativo, fornire loro le opportune indicazioni.

**2.12. Terapie complementari**

2.12.1. Informare le persone preoccupate per la loro fertilità che non vi sono sufficienti evidenze sull’efficacia delle terapie complementari per problemi di fertilità.

**2.13. Acido folico**

2.13.1. Informare le donne che cercano una gravidanza che una supplementazione di acido folico prima del concepimento e fino alla 12^ma^ settimana di gestazione riduce il rischio di difetti del tubo neurale nel bambino. La dose raccomandata è 0.4 mg/die. Raccomandare alle donne che hanno precedentemente avuto un bambino con un difetto del tubo neurale o in terapia con farmaci anti-epilettici o diabetiche una dose di 5 mg/die.

**2.14. Pregressi tagli cesarei**

2.14.1. Informare le donne che hanno avuto un precedente parto mediante taglio cesareo che, al fine di ridurre il rischio di rottura d’utero, sarebbe opportuno aspettare almeno 10 mesi dal parto precedente prima di ricominciare la ricerca di prole. **[Nuova 2020]**[[5]](#footnote-6)

**2.15. Definire l’infertilità**

2.15.1. Fornire una valutazione iniziale alle persone preoccupate a causa del ritardo del concepimento. Indagare in particolare lo stile di vita e le abitudini sessuali al fine di identificare i soggetti con minori probabilità di concepire.

2.15.2. Fornire alle coppie che non riescono ad avere rapporti sessuali o che hanno difficoltà ad averli una specifica consulenza per discutere le possibili soluzioni.

2.15.3. Il contesto in cui viene svolta la consulenza per problemi di fertilità dovrebbe consentire alle persone di discutere questioni delicate come, ad esempio, l’abuso sessuale, il maltrattamento e la violenza.

2.15.4. L’infertilità deve essere definita come una durata temporale di ricerca di gravidanza senza successo, durata che deve essere adattata alle specifiche caratteristiche della coppia. Considerare accertamenti e trattamenti solo dopo la fine di tale periodo.

2.15.5. In caso di mancato concepimento dopo un anno di rapporti sessuali vaginali non protetti, in assenza di cause note di infertilità, sottoporre le donne in età riproduttiva e i loro partner a valutazioni cliniche ed accertamenti.

2.15.6. La donna senza fattori di rischio per infertilità e che sta eseguendo un programma di inseminazioni intrauterine per problematiche sessuologiche, infettivologiche o con seme di donatore dovrebbe sottoporsi a ulteriori valutazioni cliniche e diagnostiche dopo 6 cicli con esito negativo. Nel caso venga utilizzato liquido seminale del partner, le indagini cliniche e diagnostiche più approfondite dovrebbero essere estese anche a quest’ultimo.

2.15.7. Anticipare la consulenza specialistica nei seguenti casi:

- donne di età >35 anni.
- presenza di una causa nota di infertilità o di fattori predisponenti ad una condizione di infertilità.

2.15.8. Fornire una consulenza specialistica tempestiva ai pazienti che devono sottoporsi a trattamenti che riducono la fertilità (es. terapie oncologiche).

2.15.9. Indirizzare i pazienti preoccupati per la loro fertilità con infezioni croniche (epatite B, epatite C, HIV) a centri con servizi ed esperienza adeguati per fornire indagini e trattamenti che riducano i rischi.

## 3. Diagnosi di infertilità e strategie terapeutiche

**3.1. Esame seminale**

3.1.1. L’esame del liquido seminale è parte della valutazione iniziale. I risultati devono riferirsi agli standard di riferimento emanati dall’Organizzazione Mondiale della Sanità:

- volume ≥1.5 ml.
- pH ≥7.2.
- concentrazione spermatozoi ≥15 milioni/ml.
- numero totale spermatozoi ≥39 milioni/eiaculato.
- motilità totale (percentuale della motilità progressiva e non-progressiva) ≥40% oppure motilità progressiva ≥32%.
- vitalità ≥58%.
- morfologia spermatozoi (percentuale di forme normali) ≥4%.

3.1.2. Non effettuare lo screening per gli anticorpi anti-spermatozoi perché non ci sono evidenze di un trattamento efficace per migliorare la fertilità.

3.1.3. Se i risultati del primo esame del liquido seminale non rientrano nei valori di riferimento, ripetere l’esame.

3.1.4. L’esame seminale di controllo deve essere svolto 3 mesi dopo il primo test per garantire un ciclo completo di spermatogenesi. In caso di deficit severo (azoospermia o grave oligozoospermia), ripetere l’esame di conferma il prima possibile.

**3.2. Test post-coitale del muco cervicale**

3.2.1. Non effettuare il test post-coitale del muco cervicale per valutare le coppie con problemi di fertilità perché non ha valore predittivo sulla probabilità di gravidanza.

**3.3. Infezione da Papillomavirus (HPV)**

3.3.1. Anche se esistono evidenze per una associazione tra HPV e infertilità maschile e femminile, non vi è un’indicazione a eseguire di routine questo test in quanto, sulla base delle evidenze oggi disponibili, l’eventuale positività al test non modifica il percorso terapeutico delle coppie. **[Nuova 2020]**[[6]](#footnote-7),[[7]](#footnote-8)

**3.4. Test di riserva ovarica**

3.4.1. Considerare l'età della donna come fattore predittivo di concepimento spontaneo.

3.4.2. Considerare l'età della donna come fattore predittivo di successo della fecondazione *in vitro* (Figura 1).

***Figura 1:*** Distribuzione delle probabilità di gravidanza cumulative (e relativi intervalli di confidenza al 95%) per ciclo iniziato con ovociti propri per classe di età (Dati Registro PMA – 2017). Disponibile a: [*http://old.iss.it/binary/rpma/cont/C_17_pubblicazioni_2866_allegato.pdf*](http://old.iss.it/binary/rpma/cont/C_17_pubblicazioni_2866_allegato.pdf)

3.4.3. Come fattore predittivo della risposta ovarica alla stimolazione con gonadotropine durante la fecondazione *in vitro* utilizzare una delle seguenti valutazioni:

- conta dei follicoli antrali (AFC) totale ≤4 per predire una scarsa risposta e ≥16 per una risposta elevata.
- ormone anti-Mülleriano (AMH) ≤5.4 pmol/l (0.75 ng/ml) per predire una scarsa risposta e ≥25 pmol/l (3.5 ng/ml) per una risposta elevata.

Nei casi di sospetta grave compromissione della riserva ovarica, valutare come completamento delle indagini il dosaggio dell’ormone follicolo-stimolante (FSH) e dell’estradiolo (E2) in fase follicolare precoce.

3.4.4. Non utilizzare singolarmente nessuno dei seguenti test per predire l'esito di un trattamento per la fertilità:

- volume ovarico.
- flusso sanguigno ovarico.
- inibina B.
- estradiolo (E2).

**3.5. Regolarità dei cicli mestruali**

3.5.1. Nelle donne preoccupate per la loro fertilità indagare la frequenza e la regolarità dei cicli mestruali. Informare le donne con cicli mestruali regolari che i loro cicli sono verosimilmente ovulatori.

3.5.2. Alle donne che si stanno sottoponendo ad indagini per l'infertilità richiedere il dosaggio serico di progesterone in fase medio-luteale del ciclo (il 21° giorno di un ciclo di 28 giorni) per confermare l'avvenuta ovulazione anche in presenza di cicli mestruali regolari.

3.5.3. Alle donne con cicli mestruali di durata superiore a 28 giorni richiedere il dosaggio serico di progesterone. In base alla lunghezza dei cicli mestruali, questo test potrebbe dover essere effettuato più tardivamente (per esempio il 28° giorno di un ciclo di 35 giorni) e ripetuto una volta a settimana fino all'inizio del ciclo mestruale successivo.

3.5.4. L'uso delle curve della temperatura corporea basale per confermare l'ovulazione non è raccomandato in quanto non affidabile.

3.5.5. Alle donne con cicli mestruali irregolari prescrivere il dosaggio serico di FSH e ormone luteinizzante (LH) in fase follicolare precoce.

**3.6. Dosaggio della prolattina**

3.6.1. Alle donne preoccupate per la loro fertilità non prescrivere il dosaggio serico della prolattina. Prescrivere questo test esclusivamente a donne con anovulatorietà, galattorrea o tumore ipofisario.

**3.7. Test di funzionalità tiroidea**

3.7.1. Considerato che le donne con problemi di fertilità non sono maggiormente soggette a patologie tiroidee rispetto alla popolazione generale, non prescrivere di routine i test di funzionalità tiroidea.

**3.8. Biopsia endometriale**

3.8.1. Non effettuare la biopsia endometriale e esame istologico per valutare la fase luteale perché non ci sono evidenze che un trattamento medico dei difetti della fase luteale aumenti le probabilità di gravidanza.

**3.9. Valutazione delle anomalie tubariche e uterine**

3.9.1. Nelle donne senza comorbidità note (es. malattia infiammatoria pelvica, pregressa gravidanza ectopica o endometriosi) effettuare l'isterosalpingografia (HSG) per valutare la pervietà tubarica. È un test affidabile per escludere un'occlusione tubarica ed è meno invasivo e più costo-efficace rispetto alla laparoscopia. In Centri con adeguata competenza, la valutazione ecografica della pervietà tubarica (isterosonosalpingografia) può essere considerata una valida alternativa alla HSG.

3.9.2. Eseguire la laparoscopia con salpingocromoscopia solo nelle donne con sospette comorbidità per valutare contemporaneamente la patologia tubarica e la presenza di altre patologie pelviche.

3.9.3. Il riscontro di impervietà tubarica monolaterale prossimale non ha un impatto sulla probabilità di successo delle IUI. Le pazienti in questa situazione devono essere considerate alla stregua delle pazienti con pervietà bilaterale. Al contrario, il riscontro di un’occlusione distale si associa ad un dimezzamento delle probabilità di successo. **[Nuova 2020]**[[8]](#footnote-9)

3.9.4. L’isteroscopia di routine non fa parte degli esami per la valutazione iniziale della donna.

**3.10. Test microbiologici**

3.10.1. Alle persone che si sottopongono a trattamenti fecondazione *in vitro* prescrivere i test per Sifilide, HIV, epatite B ed epatite C come da indicazioni in materia DLGS 16/2010 e successive integrazioni (compreso DLGS 85/2012 e DPR 131/2019) (per le inseminazioni da donatore cfr. *raccomandazione 4.3.1*).

3.10.2. Alle persone con infezione da HIV, epatite B o epatite C fornire una consulenza specialistica specifica e un appropriato trattamento clinico dell’infezione.

**3.11. Trasmissione virale**

3.11.1. Per le coppie in cui l'uomo è affetto da infezione da HIV, per le decisioni sulla gestione della fertilità coinvolgere nella discussione la coppia, uno specialista in fertilità e un infettivologo esperto in HIV.

3.11.2. Informare le coppie in cui l'uomo è affetto da infezione da HIV che il rischio di trasmissione del virus HIV alla donna tramite i rapporti sessuali non protetti è trascurabile quando vengono soddisfatti tutti i seguenti criteri:

- l'uomo assume regolarmente la terapia antiretrovirale altamente attiva (HAART)
- l'uomo ha una carica virale plasmatica <50 copie/ml da oltre 6 mesi
- non sono presenti altre infezioni genitali
- i rapporti non protetti sono limitati al periodo dell'ovulazione.

3.11.3. Informare le coppie che, se sono soddisfatti tutti i criteri della *raccomandazione 3.11.2*, il trattamento per la riduzione della carica virale sul liquido seminale non riduce ulteriormente il rischio di infezione ma può diminuire la probabilità di gravidanza.

3.11.4. Nelle coppie in cui l'uomo è affetto da infezione da HIV e non assume regolarmente la HAART o la sua carica virale plasmatica è >50 copie/ml, eseguire il trattamento per la riduzione della carica virale sul liquido seminale.

3.11.5. Informare le coppie che il trattamento per la riduzione della carica virale del liquido seminale riduce, ma non elimina, il rischio di trasmissione di HIV.

3.11.6. Per le coppie che soddisfano tutti i criteri della *raccomandazione 3.11.2* ma percepiscono come inaccettabile il rischio di trasmissione del virus anche dopo discussione con lo specialista in HIV, considerare il trattamento per la riduzione della carica virale del liquido seminale.

3.11.7. Informare le coppie che non ci sono sufficienti evidenze per raccomandare alle donne HIV-negative di effettuare la profilassi pre-esposizione, quando sono soddisfatti tutti i criteri della *raccomandazione 3.11.2*.

3.11.8. Eseguire la vaccinazione prima di iniziare i trattamenti per l’infertilità nei partner di soggetti con epatite B.

3.11.9. Non effettuare il trattamento per la riduzione della carica virale del liquido seminale come parte di un trattamento per l’infertilità a uomini affetti da epatite B.

3.11.10. Per le coppie in cui l'uomo è affetto da infezione da epatite C, per le decisioni sulla gestione dell’infertilità, coinvolgere nella discussione la coppia, uno specialista in fertilità, e uno esperto in epatite C.

3.11.11. Informare le coppie che vogliono concepire e nelle quali l'uomo è affetto da epatite C che il rischio di trasmissione attraverso rapporti sessuali non protetti è basso.

3.11.12. Per gli uomini con epatite C discutere con uno specialista le opzioni terapeutiche per eradicare la malattia prima di considerare il concepimento.

**3.12. Recettività alla rosolia**

3.12.1. Alle donne preoccupate per la loro fertilità prescrivere il test per valutare la recettività alla rosolia al fine di identificare quelle da sottoporre a vaccinazione. Sottoporre a vaccinazione le donne recettive alla rosolia informandole che il concepimento è sconsigliato per almeno 1 mese dopo la vaccinazione.

**3.13. Screening per il carcinoma della cervice**

3.13.1. Per evitare ritardi nei trattamenti dell’infertilità, alle donne preoccupate per la loro fertilità chiedere la data e il risultato del più recente test di screening per carcinoma della cervice uterina. Effettuare lo screening in conformità a quanto previsto dai servizi sanitari regionali.

## 4. Trattamento medico e chirurgico dell’infertilità maschile

**4.1. Trattamento medico**

4.1.1. Trattare i pazienti affetti da ipogonadismo ipogonadotropo con gonadotropine perché si sono dimostrate efficaci nel migliorare la fertilità.

4.1.2. Nei pazienti con anomalie del liquido seminale da causa inspiegata e livelli bassi di FSH periferico, non prescrivere anti-estrogeni, androgeni, bromocriptina o farmaci che aumentano i livelli di chinina perché non sono efficaci. L’impiego delle gonadotropine potrebbe migliorare la qualità del liquido seminale ma le evidenze sono insufficienti per consigliarne l’uso sistematico.

4.1.3. Informare i pazienti che il significato clinico della positività ad anticorpi anti-spermatozoi è incerto così come l’uso dei corticosteroidi sistemici come terapia.

4.1.4. Non prescrivere ai pazienti con riscontro di leucospermia una terapia antibiotica se non in presenza di un’accertata infezione, dal momento che non esistono evidenze che questo aumenti la probabilità di gravidanza.

**4.2. Trattamento chirurgico**

4.2.1. La correzione chirurgica dell’azoospermia dovuta a ostruzione epididimale dovrebbe essere proposta in Centri con adeguata competenza dal momento che questo intervento è potenzialmente in grado di rendere pervio il dotto e aumentare la fertilità. Considerare la correzione chirurgica come una valida alternativa al recupero chirurgico degli spermatozoi e alle successive tecniche di fecondazione *in vitro*.

4.2.2. Considerare il trattamento chirurgico del varicocele per incrementare le probabilità di concepimento naturale in uomini infertili con alterazioni del liquido seminale, varicocele clinico e partner di età inferiore a 35 anni e con buona riserva ovarica. **[Aggiornata 2020][[9]](#footnote-10)**

4.2.3. Considerare il trattamento chirurgico per varicocele clinico nei casi di infertilità maschile con indicazione a fecondazione *in vitro*, ivi compresi i casi di azoospermia non ostruttiva. **[Nuova 2020]**[[10]](#footnote-11),[[11]](#footnote-12)

**4.3. Trattamento del difetto di eiaculazione**

4.3.1. Il trattamento dei problemi di eiaculazione può ripristinare la fertilità senza dover ricorrere a metodi invasivi per il recupero degli spermatozoi o tecniche di fecondazione assistita. Tuttavia, sono necessarie ulteriori studi per meglio valutare le opzioni terapeutiche disponibili.

## 5. Disturbi dell’ovulazione

**Classificazione dei disturbi dell’ovulazione**

L’Organizzazione Mondiale della Sanità (OMS) classifica i disturbi dell’ovulazione in tre gruppi:

- Gruppo I: insufficienza ipotalamo-ipofisaria (amenorrea ipotalamica o ipogonadismo ipogonadotropo).
- Gruppo II: disfunzione ipotalamo-ipofisi-ovarica (prevalentemente sindrome dell’ovaio policistico).
- Gruppo III: insufficienza ovarica.

**5.1. Disturbi dell’ovulazione del Gruppo I**

5.1.1. Al fine di incrementare le probabilità di ovulazione, concepimento e normale gravidanza consigliare alle pazienti con disturbo ovulatorio del Gruppo I:

- aumento del peso corporeo in caso di BMI <19 e/o
- riduzione dell’esercizio fisico se si stanno sottoponendo a esercizio fisico intenso.

5.1.2. Prescrivere alle pazienti con disturbo ovulatorio del Gruppo I la somministrazione di gonadotropine con attività LH per indurre l’ovulazione.

**5.2. Disturbi dell’ovulazione del Gruppo II**

Nelle pazienti con disturbo ovulatorio del Gruppo II sottoposte a trattamenti di prima linea per la stimolazione ovarica:

5.2.1. Consigliare la perdita di peso alle pazienti con BMI ≥30 (cfr. *raccomandazione 2.6*). Informarle del fatto che la sola riduzione del peso può ripristinare l’ovulazione, migliorare la risposta all’induzione dell’ovulazione e influenzare positivamente gli outcome della gravidanza.

5.2.2. Prescrivere uno dei seguenti trattamenti, tenendo in considerazione potenziali effetti avversi, facilità e modalità d’uso, BMI e necessità di monitoraggio:

- Clomifene citrato *o*
- Metformina (solo off-label) **[Aggiornata 2020]**[[12]](#footnote-13)

5.2.3. Sottoporre le pazienti in terapia con clomifene citrato ad un monitoraggio ecografico, almeno al primo ciclo di trattamento, per verificare che stiano assumendo una dose tale da minimizzare il rischio di gravidanza multipla.

5.2.4. Per le pazienti in terapia con clomifene citrato, non protrarre il trattamento oltre i 6 mesi.

5.2.5. Informare le pazienti in terapia con metformina degli effetti collaterali associati al suo uso (nausea, vomito e altri disturbi gastrointestinali).

**Nelle pazienti con disturbo ovulatorio del Gruppo II resistenti al trattamento con clomifene citrato:**

5.2.6. Considerare uno dei seguenti trattamenti di seconda linea, tenendo conto delle caratteristiche cliniche delle pazienti e delle loro preferenze:

- Trattamento combinato con clomifene citrato e metformina.
- Gonadotropine. **[Aggiornata 2020]**[[13]](#footnote-14)

5.2.7. Non prescrivere alle pazienti con sindrome dell’ovaio policistico in terapia con gonadotropine una terapia concomitante con GnRH agonisti, dal momento che non aumenta la probabilità di gravidanza ma aumenta il rischio di sindrome da iperstimolazione ovarica.

5.2.8. L’impiego adiuvante di ormone della crescita (GH) in corso di stimolazione dell’ovulazione non è raccomandato perché non aumenta la probabilità di gravidanza.

**5.3. Amenorrea iperprolattinemica – dopamino agonisti**

5.3.1. Alle pazienti con disturbi ovulatori causati da iperprolattinemia prescrivere un trattamento con dopamino-agonisti. Nella scelta del farmaco considerare la sicurezza del suo uso in gravidanza e la minimizzazione dei costi.

**5.4. Monitoraggio dell’induzione dell’ovulazione durante la terapia con gonadotropine**

5.4.1. Prima dell’inizio del trattamento, informare le pazienti a cui viene prescritta la terapia con gonadotropine dei rischi di gravidanza multipla e di sindrome da iperstimolazione ovarica.

5.4.2. Il monitoraggio ecografico per la misurazione del diametro follicolare e il numero di follicoli in crescita deve essere parte integrante della terapia con gonadotropine, al fine di ridurre i rischi di gravidanza multipla e di sindrome d iperstimolazione ovarica.

## 6. Chirurgia tubarica e uterina

**6.1. Chirurgia tubarica**

6.1.1. In donne con patologia tubarica di modesta gravità, la chirurgia tubarica può risultare efficace. Nei centri con adeguata expertise, la chirurgia tubarica può essere considerata come un’opzione terapeutica.

**6.2. Chirurgia per idrosalpingi prima del trattamento di fecondazione in vitro**

6.2.1. Prima del trattamento di fecondazione in vitro, alle pazienti con idrosalpinge dovrebbe essere suggerito un intervento di salpingectomia, preferibilmente per via laparoscopica, per aumentare le probabilità di successo.

**6.3. Chirurgia uterina**

6.3.1. Nelle pazienti con amenorrea da sinechie endouterine effettuare un intervento isteroscopico di adesiolisi, in quanto può determinare un ripristino del ciclo mestruale e aumentare le probabilità di gravidanza.

## 7. Endometriosi

Per quanto attinente al trattamento dell’infertilità associata ad endometriosi si rimanda alle specifiche linee guida dedicate alla malattia.

## 8. Infertilità inspiegata

**8.1. Stimolazione ovarica per il trattamento dell’infertilità inspiegata**

8.1.1. Non prescrivere farmaci per la stimolazione ovarica per via orale (clomifene citrato) alle pazienti con infertilità inspiegata.

8.1.2. Informare le pazienti con infertilità inspiegata che la sola stimolazione dell’ovulazione con clomifene citrato non aumenta le probabilità di gravidanza.

8.1.3. Consigliare alle pazienti con infertilità inspiegata che hanno rapporti regolari non protetti di cercare di concepire per almeno 2 anni prima di considerare la fecondazione *in vitro* (incluso un periodo fino ad 1 anno precedente alle indagini per infertilità). Nelle donne di età >35 anni considerare la fecondazione *in vitro* prima dei 2 anni. **[Aggiornata 2020]**

8.1.4. Prescrivere la fecondazione *in vitro* (cfr. *raccomandazione 11.1.3 e 11.1.4*) alle pazienti con infertilità inspiegata che non hanno concepito dopo 2 anni di rapporti regolari non protetti (incluso un periodo fino ad 1 anno precedente alle indagini per infertilità). Nelle donne di età >35 anni considerare la fecondazione *in vitro* prima dei 2 anni. **[Aggiornata 2020]**

## 9. Inseminazione intrauterina

**9.1. Inseminazione intrauterina**

9.1.1. Considerare l’inseminazione intrauterina senza stimolazione ovarica come opzione terapeutica alternativa ai rapporti sessuali non protetti nei seguenti gruppi:

- Soggetti che non riescono o hanno difficoltà ad avere rapporti sessuali vaginali completi per disabilità fisiche o problematiche psicosessuali.
- Donazione di seme.
- Soggetti affetti da condizioni che richiedono particolare attenzione riguardo al metodo di concepimento (es. necessità di trattamento per la riduzione della carica virale sul liquido seminale laddove il partner sia HIV positivo).

9.1.2. Per le donne di cui al *punto 9.1.1*. che non abbiano concepito dopo 6 cicli di inseminazione, nonostante un’ovulazione regolare, una dimostrata pervietà tubarica ed un esame del liquido seminale normale, considerare ulteriori 6 cicli di inseminazione intrauterina oppure proporre la fecondazione *in vitro*.

9.1.3. Per i casi di infertilità inspiegata, endometriosi lieve o infertilità da fattore maschile lieve, in coppie con rapporti sessuali regolari non protetti:

- Non prescrivere di routine l’inseminazione intrauterina, con o senza stimolazione ovarica (se non in casi eccezionali come, ad esempio, rifiuto della fecondazione in vitro per motivi sociali, culturali o religiosi)
- Suggerire alla coppia di proseguire la ricerca di una gravidanza mediante rapporti sessuali non protetti per almeno 2 anni prima di considerare la fecondazione in vitro (incluso un periodo fino a 1 anno precedente alle indagini per infertilità). Nelle donne di età >35 anni considerare le tecniche di fecondazione *in vitro* prima dei 2 anni. **[Aggiornata 2020]**

## 10. Fattori predittivi di successo della fecondazione in vitro

**10.1. Età della donna**

10.1.1. Informare le pazienti che le probabilità di avere un figlio con la fecondazione in vitro si riducono progressivamente all’aumentare dell’età della donna (Figura 1).

**10.2. Numero di precedenti cicli di trattamento**

10.2.1. Informare le pazienti che la probabilità di avere un figlio con la fecondazione in vitro si riduce progressivamente all’aumentare del numero di cicli falliti.

**10.3. Precedenti gravidanze**

10.3.1. Informare le pazienti del fatto che i trattamenti di fecondazione *in vitro* risultano più efficaci in donne che abbiano già avuto gravidanze e/o figli.

**10.4. Indice di massa corporea**

10.4.1. Informare le pazienti del fatto che il BMI della donna dovrebbe essere idealmente compreso tra 19 e 29.9 prima di iniziare un percorso di fecondazione assistita e che un BMI al di fuori di questo range riduce le probabilità di successo del trattamento.

**10.5. Stile di vita**

10.5.1. Informare i pazienti del fatto che un consumo di alcol >1 unità/die riduce le probabilità di successo della fecondazione *in vitro*.

10.5.2. Informare i pazienti del fatto che il fumo da parte di entrambi i partner può ridurre i tassi di successo della fecondazione *in vitro*.

10.5.3. Informare le pazienti del fatto che l’eccesso di caffeina da parte della donna può ridurre i tassi di successo della fecondazione *in vitro*.

10.5.4. Informare i pazienti che l’attività fisica della donna prima della fecondazione *in vitro* può aumentarne i tassi di successo. **[Nuova 2020]**[[14]](#footnote-15)

## 11. Criteri di ricorso alla fecondazione *in vitro*

**11.1. Criteri di ricorso alla fecondazione *in vitro***

11.1.1. Quando si prende in considerazione la fecondazione *in vitro* discutere con la coppia rischi e benefici, adattandoli alla condizione clinica della coppia. **[Aggiornata 2020]**

11.1.2. Informare la coppia che normalmente un ciclo completo di fecondazione *in vitro*, con o senza iniezione intracitoplasmatica di spermatozoo (ICSI), comprende un ciclo di stimolazione ovarica e il successivo trasferimento degli embrioni ottenuti (considerando sia quelli trasferiti a fresco che quelli trasferiti dopo crioconservazione).

11.1.3. In donne di età <43 anni, si raccomanda di eseguire fino a 3 cicli completi di fecondazione *in vitro*, con o senza ICSI.

Per le donne tra 40 e 42 anni, raccomandare i cicli solo se sono soddisfatti i seguenti criteri aggiuntivi.

- Non evidenze di riserva ovarica ridotta.
- Discussione approfondita delle implicazioni aggiuntive della fecondazione in vitro e della gravidanza a questa età.

Se la donna dovesse compiere 43 anni durante tale programma terapeutico, completare il ciclo in corso ma non eseguirne altri.

11.1.4. In casi selezionati a particolare buona prognosi (come donne giovani che hanno già ottenuto una gravidanza), è possibile procedere con i tentativi oltre quanto esplicitato nella raccomandazione 11.1.3. Tale decisione deve essere però vagliata attentamente e giustificata prestando massima attenzione al rapporto rischi-benefici.

11.1.5. Laddove gli accertamenti dimostrino l’impossibilità di ottenere una gravidanza con una condotta d’attesa e indichino che la fecondazione *in vitro* sia l’unico trattamento efficace, inviare la coppia a un centro di riferimento per eseguire la fecondazione *in vitro*.

11.1.6. Il computo totale dei cicli di fecondazione *in vitro* deve tenere in considerazione sia quelli svolti in regime di Sistema Sanitario Nazionale sia quelli svolti in regime libero-professionale in quanto i limiti riportati sono stati definiti in base ad analisi rischi-benefici per la salute.

11.1.7. Nel valutare l’efficacia e la sicurezza di un nuovo trattamento di fecondazione *in vitro*, considerare anche l’esito dei pregressi trattamenti di fecondazione *in vitro*.

11.1.8. Definire “sospeso” un ciclo di fecondazione *in vitro* in tutti i casi in cui non viene effettuato il prelievo ovocitario. Nel valutare l’idoneità della paziente ad ulteriori cicli di fecondazione *in vitro* tenere in considerazione i precedenti cicli sospesi a seguito di insufficiente risposta alla stimolazione ovarica.

## 12. Procedure utilizzate nel trattamento di fecondazione *in vitro*

**12.1. Procedure utilizzate nel trattamento di fecondazione *in vitro***

12.1.1. Informare le donne che l’impiego di un pre-trattamento (con contraccettivi orali o progestinici) come parte della fecondazione *in vitro* non influenza le probabilità di avere un figlio.

12.1.2. Prendere in considerazione un pre-trattamento (con contraccettivi orali o progestinici) al fine di programmare il ciclo di fecondazione *in vitro*.

**12.2. Desensibilizzazione ipofisaria** **e altri protocolli per evitare il picco prematuro di LH nella fecondazione *in vitro***

12.2.1. Utilizzare protocolli per evitare il picco prematuro di LH nei cicli di fecondazione *in vitro* con stimolazione ovarica mediante gonadotropine.

12.2.2. Nei cicli di fecondazione *in vitro* con stimolazione ovarica mediante gonadotropine utilizzare GnRH agonisti o GnRH antagonisti.

12.2.3. Considerare i GnRH agonisti solo in donne con basso rischio di sindrome da iperstimolazione ovarica.

12.2.4. Quando si utilizzano GnRH agonisti per un trattamento di fecondazione *in vitro*, impiegare un protocollo lungo di desensibilizzazione ipofisaria (long protocol).

**12.3 Stimolazione ovarica nella fecondazione *in vitro***

12.3.1. Eseguire la stimolazione ovarica come parte del trattamento di fecondazione *in vitro*.

12.3.2. Utilizzare gonadotropine urinarie o ricombinanti per la stimolazione ovarica.

12.3.3. Quando si utilizzano le gonadotropine per la stimolazione ovarica nel trattamento di fecondazione *in vitro*:

- Considerare un dosaggio di gonadotropine personalizzato, sulla base di fattori predittivi di successo del trattamento, come:
  - Età
  - BMI
  - Presenza di ovaio policistico
  - Riserva ovarica
- Non utilizzare un dosaggio di gonadotropine >300 UI/die. **[Aggiornata 2020]**[[15]](#footnote-16)

12.3.4. Sottoporre le donne a un monitoraggio ecografico (eventualmente associato al dosaggio dei livelli di estradiolo e progesterone) per valutare efficacia e sicurezza durante il ciclo di stimolazione ovarica.

12.3.5. Informare le donne del fatto che i cicli di fecondazione *in vitro* con stimolazione ovarica con gonadotropine presentano tassi di gravidanza superiori rispetto alla fecondazione *in vitro* su cicli naturali.

12.3.6. Considerare la fecondazione *in vitro* su cicli naturali solo in casi attentamente selezionati.

12.3.7. Non utilizzare l’ormone della crescita come trattamento adiuvante nei protocolli di fecondazione in vitro. **[Aggiornata 2020]**[[16]](#footnote-17)

12.3.8. Considerare la somministrazione di mio-inositolo prima della fecondazione *in vitro* in quanto riduce la dose totale di gonadotropine impiegate. **[Nuova 2020]**[[17]](#footnote-18),[[18]](#footnote-19)

**12.4. Induzione dell’ovulazione nella fecondazione *in vitro***

12.4.1. Prescrivere alle donne la gonadotropina corionica umana (urinaria o ricombinante) per indurre l’ovulazione nel trattamento di fecondazione *in vitro*.

12.4.2. Considerare il trigger con GnRH agonista nei pazienti a rischio di Sindrome da Iperstimolazione ovarica. **[Nuova 2020]**[[19]](#footnote-20)

12.4.3. Eseguire il monitoraggio ecografico della risposta ovarica come parte integrante dei cicli di trattamento di fecondazione *in vitro*.

12.4.4. I centri che utilizzano gonadotropine per la stimolazione ovarica devono disporre di protocolli per la prevenzione, diagnosi e gestione della sindrome da iperstimolazione ovarica.

12.4.5. Considerare l’impiego di Agonisti della Dopamina per prevenire la sindrome da iperstimolazione ovarica in pazienti a rischio. **[Nuova 2020]**[[20]](#footnote-21)

**12.5. Prelievo ovocitario e raccolta del seme nella fecondazione *in vitro***

12.5.1. Nel corso di un prelievo ovocitario, impiegare modalità efficaci di contenimento del dolore.

12.5.2. Per una somministrazione sicura dei farmaci sedativi fare riferimento alle specifiche linee guida anestesiologiche.

12.5.3. Non eseguire il lavaggio della cavità follicolare, perché non aumenta il numero di ovociti prelevati o i tassi di gravidanza, mentre si associa ad una maggiore durata del prelievo e del dolore. **[Aggiornata 2020]**[[21]](#footnote-22)

12.5.4. La raccolta degli spermatozoi per via chirurgica prima di una ICSI può essere eseguita secondo diverse tecniche, in base alla patologia e alle preferenze del paziente. In tutti i casi, garantire la disponibilità di strutture per la criopreservazione degli spermatozoi.

12.5.5. L’*assisted hatching* su embrioni ottenuti da ciclo a fresco non è raccomandato in quanto non vi sono evidenze che aumenti i tassi di gravidanza. Le evidenze su materiale crioconservato sono ancora insufficienti. **[Aggiornata 2020]**[[22]](#footnote-23)

**12.6. Strategie di trasferimento embrionario nella fecondazione *in vitro***

12.6.1. Nelle donne che si sottopongono a trattamenti di fecondazione *in vitro* eseguire il trasferimento embrionario eco-assistito perché aumenta i tassi di gravidanza.

12.6.2. Il trasferimento embrionario in presenza di uno spessore endometriale <5 mm difficilmente esita in una gravidanza e pertanto non è raccomandato.

12.6.3. Informare le donne che il riposo a letto per più di 20 minuti dopo il trasferimento embrionario non migliora l’esito del trattamento di fecondazione *in vitro*.

12.6.4. Utilizzare le raccomandazioni dell’ESHRE per valutare lo stato di sviluppo degli embrioni, sia allo stadio di clivaggio che a quello di blastocisti. **[Aggiornata 2020]**[[23]](#footnote-24)

12.6.5. Nel valutare il numero di embrioni freschi o crioconservati da trasferire, seguire le seguenti indicazioni:

- Per donne di età <37 anni:
- Nel primo ciclo completo di fecondazione *in vitro* trasferire un singolo embrione.
- Nel secondo ciclo completo di fecondazione *in vitro* se si dispone di 1 o più embrioni di ottima qualità morfologica trasferire un singolo embrione. Considerare il trasferimento di 2 embrioni se non sono disponibili embrioni di ottima qualità morfologica.
- Nel terzo ciclo completo di fecondazione *in vitro* trasferire non più di 2 embrioni.
- Per donne di età compresa tra 37 e 39 anni:
- Nel primo e secondo ciclo completo di fecondazione *in vitro* se si dispone di 1 o più embrioni di ottima qualità morfologica trasferire un singolo embrione. Considerare il trasferimento di 2 embrioni se non sono disponibili embrioni di ottima qualità morfologica.
- Nel terzo ciclo completo di fecondazione *in vitro* trasferire non più di 2 embrioni.
- Per donne di età compresa tra i 40 e 42 anni considerare il trasferimento di 2 embrioni.

12.6.6. Per le donne che si sottopongono ad un trattamento di fecondazione *in vitro* con ovociti da donatrice, scegliere la strategia di trasferimento embrionario come riportato alla *raccomandazione 12.6.5*. per le donne di età < 37 anni. Prestare attenzione nella scelta anche alla salute generale della ricevente e ai suoi possibili rischi ostetrici.

12.6.7. Non trasferire più di 2 embrioni in nessun ciclo di fecondazione *in vitro*.

12.6.8. Se è disponibile una blastocisti di ottima qualità morfologica, trasferire un solo embrione.

12.6.9. Quando si prende in considerazione il trasferimento di 2 embrioni, informare le coppie dell’associato rischio di gravidanza multipla.

12.6.10. Informare le donne che il trasferimento di un solo embrione non protegge in assoluto contro la gravidanza gemellare: la fecondazione *in vitro* si associa a un rischio 2-3 volte superiore di gravidanza monozigote. **[Nuova 2020]**[[24]](#footnote-25),[[25]](#footnote-26)

12.6.11. Informare le donne che la fecondazione *in vitro* si associa ad un rischio aumentato di placentazione anomala (placenta previa, distacco di placenta, inserzioni anomale del funicolo). **[Nuova 2020]**[[26]](#footnote-27)

12.6.12. Garantire la criopreservazione degli embrioni non trasferiti.

12.6.13. Informare le donne con cicli ovulatori regolari che le probabilità di gravidanza dopo il trasferimento di un embrione criopreservato e scongelato sono simili per embrioni trasferiti su un ciclo spontaneo o su un ciclo da terapia ormonale sostitutiva.

12.6.14. Informare le donne della possibilità di effettuare la diagnosi preimpianto per l'identificazione di aneuploidie embrionali ma che questa tecnica non può aumentare le probabilità di gravidanza. **[Nuova 2020]**[[27]](#footnote-28),[[28]](#footnote-29),[[29]](#footnote-30)

**12.7. Supporto della fase luteale dopo fecondazione *in vitro***

12.7.1. Prescrivere alle donne progesterone per il supporto della fase luteale dopo un trattamento di fecondazione *in vitro*.

12.7.2. Non prescrivere di routine la gonadotropina corionica umana per il supporto della fase luteale dopo un trattamento di fecondazione *in vitro* in quanto aumenta il rischio di sindrome da iperstimolazione ovarica.

12.7.3. Informare le donne che si stanno sottoponendo ad un trattamento di fecondazione *in vitro* che non vi sono evidenze che giustifichino il proseguimento del supporto della fase luteale oltre le 5 settimane di gestazione. **[Aggiornata 2020]**[[30]](#footnote-31)

**12.8. Trasferimento intratubarico di gameti e trasferimento intratubarico di zigoti**

12.8.1. Non vi sono sufficienti evidenze per raccomandare il trasferimento intratubarico di gameti o di zigoti rispetto al trasferimento intrauterino di embrioni.

## 13. Iniezione intracitoplasmatica dello spermatozoo (ICSI)

**13.1. Indicazioni per la ICSI**

13.1.1. Le indicazioni riconosciute per l’uso della ICSI sono:

- Fattore maschile grave (alterazioni severe della qualità del liquido seminale).
- Azoospermia ostruttiva.
- Azoospermia non ostruttiva.
- Utilizzo di ovociti crioconservati.

In aggiunta, considerare la ICSI in coppie in cui in un precedente ciclo di trattamento con fecondazione *in vitro* classica non sia stata osservata fecondazione o il tasso di fecondazione sia stato molto basso.

**13.2. Aspetti genetici e consulenza**

13.2.1. Prima del trattamento con ICSI per fattore maschile severo, sottoporre le coppie ad indagini appropriate sia a scopo diagnostico sia per consentire una discussione informata sulle implicazioni del trattamento.

13.2.2. Prima del trattamento con ICSI, considerare i possibili risvolti genetici.

13.2.3. Nei casi in cui è noto o sospetto un difetto genetico specifico associato con infertilità maschile, fornire alla coppia un’adeguata consulenza genetica ed idonei accertamenti genetici.

13.2.4. Nei casi in cui l’indicazione alla ICSI è un fattore maschile grave o un’azoospermia non ostruttiva, valutare il cariotipo del partner maschile.

13.2.5. Ai soggetti che si sottopongono ad indagine del cariotipo, fornire una consulenza genetica sulle anomalie che potrebbero essere riscontrate.

13.2.6. Non considerare la ricerca delle microdelezioni del cromosoma Y come indagine di *routine* prima della ICSI. Tuttavia, informare le coppie che una percentuale significativa dell’infertilità maschile deriva verosimilmente da anomalie di geni situati sul cromosoma Y, coinvolti nella regolazione della spermatogenesi.

**13.3. ICSI *versus* fecondazione *in vitro* classica**

13.3.1 Informare le coppie del fatto che con la ICSI il tasso di fecondazione potrebbe essere superiore a quello della fecondazione *in vitro* classica ma che a fecondazione avvenuta, il tasso di gravidanze non è superiore.

## 14. Indicazioni all’inseminazione da donatore

**14.1. Indicazioni all’inseminazione da donatore**

14.1.1. Eseguire l’inseminazione con spermatozoi da donatore nel caso in cui non siano disponibili gameti competenti anche dopo tentativi di recupero di spermatozoi (con tecniche chirurgiche o mediche). Se la coppia non vuole esporsi ai rischi delle procedure di recupero o a quelli della fecondazione *in vitro* con ICSI, proporre l’inseminazione con spermatozoi da donatore.

14.1.2. Considerare l’inseminazione con spermatozoi da donatore anche nelle seguenti condizioni:

- rischio elevato di trasmettere una malattia genetica alla prole.
- rischio elevato di trasmettere una malattia infettiva alla prole o alla partner.
- isoimmunizzazione Rh severa.

**14.2 Informazione**

14.2.1. Informare le coppie con fattore maschile severo in merito ai rischi e ai benefici sia della ICSI omologa che della donazione.

14.2.2. Alle coppie che prendono in considerazione l’inseminazione con spermatozoi da donatore, offrire una consulenza sulle implicazioni mediche, genetiche e psicologiche del trattamento per la coppia stessa e per la prole.

**14.3. Reclutamento**

14.3.1. I Centri di medicina della riproduzione attivi nel reclutamento di donatori e nella crioconservazione di liquido seminale devono attenersi alle norme riportate dal D. Lgs. 16/2010 e successive integrazioni (compreso D. Lgs. 85/2012 e DPR 131/2019).

14.3.2. Offrire a tutti i potenziali donatori di spermatozoi una consulenza in merito alle implicazioni del trattamento per loro stessi (comprese quelle di carattere giuridico e psicologico).

**14.4. Trattamento**

14.4.1. Prima di iniziare trattamenti con spermatozoi da donatore è importante:

- avere conferma di cicli ovulatori nella donna.
- eseguire test diagnostici mirati a valutare la pervietà tubarica in caso di storia suggestiva di danno tubarico.

14.4.2. In donne prive di fattori di rischio, non è essenziale eseguire i test di valutazione della pervietà tubarica. Considerare questi test dopo 3 cicli falliti.

14.4.3. In caso di utilizzo di spermatozoi di donatore preferire l’inseminazione intrauterina a quella cervicale poiché si associa a tassi di gravidanza più elevati.

14.4.4. Offrire fino a 6 cicli di inseminazione senza stimolazione ovarica al fine di ridurre il rischio di gemellarità.

## 15. Donazione di ovociti

**15.1. Indicazioni alla donazione di ovociti**

15.1.1. L’uso di ovociti da donatrice è indicato nelle seguenti condizioni:

- Menopausa precoce
- Disgenesie gonadiche come la Sindrome di Turner
- Ovariectomia bilaterale
- Menopausa a seguito di radio o chemioterapia
- Ripetuti fallimenti di cicli di fecondazione *in vitro* omologhi
- Non disponibilità di gameti competenti (come età avanzata)

Inoltre, la procedura può essere presa in considerazione in caso di elevato rischio di trasmissione di malattie genetiche alla prole.

**15.2. Screening delle donatrici**

15.2.1. I Centri di medicina della riproduzione attivi nel reclutamento di donatrici e nella crioconservazione di ovociti devono attenersi alle disposizioni riportate dal D. Lgs. 16/2010 e successive integrazioni (compreso D. Lgs. 85/2012 e DPR 131/2019).

**15.3. Donazione di ovociti e egg sharing**

15.3.1. Informare le donatrici di ovociti in merito ai rischi della stimolazione ovarica e del prelievo ovocitario.

15.3.2. Garantire alle donatrici e alle riceventi una consulenza sulle implicazioni a breve e lungo termine del trattamento, in ambito giuridico, medico, genetico e psicologico.

15.3.3. Offrire una consulenza specifica a tutte le coppie che considerano un programma di egg-sharing.

15.3.4. Le donne che si sottopongono a ovodonazione devono essere informate degli aumentati rischi ostetrici ed in particolare dei rischi di parto prematuro, disturbi pressori in gravidanza e di avere neonati di basso peso. La consulenza deve essere personalizzata sulla base dello specifico quadro clinico della paziente. **[Nuovo 2020]**[[31]](#footnote-32),[[32]](#footnote-33),[[33]](#footnote-34)

## 16. Pazienti oncologici che desiderano preservare la loro fertilità

16.1.1. Nei pazienti in procinto di iniziare trattamenti chemioterapici o radioterapici che possono ridurne la fertilità, considerare la crioconservazione secondo le raccomandazioni dell’AIOM sul SNLG.

16.1.2. Al momento della diagnosi di tumore, il team oncologico deve discutere con il/la paziente l’impatto della malattia e del trattamento sulla fertilità futura.

16.1.3. Nella decisione di eseguire o meno tecniche di preservazione della fertilità tenere conto dei seguenti fattori:

- età;
- diagnosi oncologica;
- piano terapeutico;
- risultati attesi dai trattamenti di preservazione della fertilità;
- prognosi del trattamento oncologico;
- impatto dei processi di congelamento, mantenimento in biobanca e scongelamento sul materiale crioconservato;
- riserva ovarica (per la donna).

16.1.4. Considerare l’impiego di strumenti di informazione e di supporto decisionale (cartacei, video o online) perché incoraggiano la partecipazione attiva dei pazienti nelle decisioni cliniche e rendono più consapevole la loro scelta. **[Nuova 2020]**[[34]](#footnote-35)

16.1.5. Per la preservazione della fertilità in pazienti oncologici, non utilizzare gli stessi criteri di accesso adottati per i convenzionali trattamenti per l’infertilità.

16.1.6. Non applicare un limite inferiore di età per la crioconservazione a scopo di preservazione della fertilità in pazienti oncologici.

16.1.7. Informare i pazienti oncologici che le norme sui criteri di accesso per i trattamenti dell’infertilità non sono applicabili per la preservazione della fertilità. Tuttavia, queste stesse norme verranno applicate quando sarà utilizzato il materiale crioconservato.

16.1.8. Quando si decide di preservare la fertilità in pazienti oncologici, crioconservare liquido seminale o ovociti o tessuto ovarico.

16.1.9. Proporre la crioconservazione del liquido seminale a uomini e adolescenti che stanno per essere sottoposti ad una terapia oncologica che probabilmente li renderà infertili.

16.1.10. Crioconservare il liquido seminale in azoto liquido.

16.1.11. Proporre la crioconservazione ovocitaria a donne in età riproduttiva (comprese le adolescenti) che stanno per essere sottoposte a trattamenti oncologici gonadotossici se:

- il loro stato di salute generale è tale da consentire loro di sottoporsi alla stimolazione ovarica controllata e al recupero ovocitario e
- tali procedure non determineranno un peggioramento del loro stato di salute e
- vi è sufficiente tempo prima delle terapie oncologiche
- hanno una riserva ovarica sufficiente.

16.1.12. Proporre la crioconservazione di tessuto ovarico in pazienti prepuberi o in giovane età che stanno per essere sottoposte a trattamenti oncologici gonadotossici se:

- il loro stato di salute generale è tale da consentire loro di sottoporsi a un intervento chirurgico e
- tali procedure non determineranno un peggioramento del loro stato di salute e
- vi è sufficiente tempo prima delle terapie oncologiche. [Nuova 2020]

16.1.13. Come tecnica di crioconservazione degli ovociti, preferire la vitrificazione al congelamento lento quando attrezzature e competenze sono disponibili.

16.1.14. Includere nel consenso informato la durata e le modalità di rinnovo della crioconservazione dei gameti. **[Aggiornata 2020]**

## 17. Sicurezza a lungo termine delle tecniche di riproduzione medicalmente assistita

**17.1. Effetti a lungo termine della stimolazione ovarica**

17.1.1. Fornire alle donne che stanno prendendo in considerazione l’induzione dell’ovulazione o la stimolazione ovarica informazioni aggiornate in merito agli effetti a lungo termine di tale trattamento sulla salute.

17.1.2. Informare le coppie a cui sono state proposte l’induzione dell’ovulazione o la stimolazione ovarica che:

- non è stata dimostrata alcuna associazione diretta tra questi trattamenti e l’insorgenza di neoplasie maligne.
- non è stata dimostrata associazione nel breve e medio termine tra questi trattamenti ed effetti avversi nei bambini nati (inclusa l’insorgenza di neoplasie maligne).
- non sono ancora disponibili informazioni in merito agli effetti a lungo termine sulla salute delle donne trattate e su quella dei bambini nati.

17.1.3. Limitare l’uso dei farmaci per l’induzione dell’ovulazione e per la stimolazione ovarica alla minima dose efficace e alla minima durata di utilizzo.

**17.2. Effetti a lungo termine e sicurezza della fecondazione *in vitro***

17.2.1. Fornire ai soggetti che stanno prendendo in considerazione un trattamento di fecondazione *in vitro*, con o senza ICSI, informazioni aggiornate in merito agli effetti a lungo termine di tali trattamenti sulla salute (inclusi i rischi legati a gravidanze multiple).

17.2.2. Informare i soggetti che stanno prendendo in considerazione un trattamento di fecondazione in vitro, con o senza ICSI che, mentre il rischio assoluto di effetti avversi a lungo termine è basso, non si può escludere un minimo aumento del rischio di tumori borderline dell’ovaio.

17.2.3. Ancorché siano eventi molto rari, informare i soggetti che stanno prendendo in considerazione un trattamento di fecondazione *in vitro*, con o senza ICSI, che i bambini nati sono ad aumentato rischio di malattie epigenetiche da difetti dell’imprinting. **[Nuova 2020]**[[35]](#footnote-36)

17.2.4. Informare i soggetti che stanno prendendo in considerazione un trattamento di fecondazione *in vitro*, con o senza ICSI, che i bambini nati hanno un modesto aumento del rischio di malformazioni rispetto alla popolazione generale**.** Non ci sono tuttavia evidenze che sia la tecnica e non la condizione di infertilità a determinare il rischio. **[Aggiornata 2020]**[[36]](#footnote-37),[[37]](#footnote-38),[[38]](#footnote-39),[[39]](#footnote-40)

1. Dunson DB, Baird DD, Colombo B. Increased infertility with age in men and women. Obstet Gynecol. 2004 Jan;103(1):51-6. [↑](#footnote-ref-2)
2. Per unità di alcol si intende: 1 bicchiere (125 ml) di vino di media gradazione, o 1 lattina di birra (330 ml) di media gradazione, o 1 bicchierino (40 ml) di superalcolico. [↑](#footnote-ref-3)
3. Lyngsø J, Ramlau-Hansen CH, Bay B, Ingerslev HJ, Hulman A, Kesmodel US. Association between coffee or caffeine consumption and fecundity and fertility: a systematic review and dose-response meta-analysis. Clin Epidemiol. 2017 Dec 15;9:699-719. [↑](#footnote-ref-4)
4. Henriques MC, Loureiro S, Fardilha M, Herdeiro MT. Exposure to mercury and human reproductive health: A systematic review. Reprod Toxicol. 2019 Apr;85:93-103. [↑](#footnote-ref-5)
5. Matorras R, Berreteaga L, Laínz L, Exposito A, Martínez L. Influence of Caesarean section-pregnancy interval on uterine rupture risk and IVF pregnancy rates: systematic review and mathematical modelling. Reprod Biomed Online. 2019 Nov;39(5):809-818. [↑](#footnote-ref-6)
6. Yuan S, Qiu Y, Xu Y, Wang H. Human papillomavirus infection and female infertility: a systematic review and meta-analysis. Reprod Biomed Online. 2020 Feb;40(2):229-237. [↑](#footnote-ref-7)
7. Weinberg M, Sar-Shalom Nahshon C, Feferkorn I, Bornstein J. Evaluation of human papilloma virus in semen as a risk factor for low sperm quality and poor in vitro fertilization outcomes: a systematic review and meta-analysis. Fertil Steril. 2020 May;113(5):955-969.e4. [↑](#footnote-ref-8)
8. Tan J, Tannus S, Taskin O, Kan A, Albert AY, Bedaiwy MA. The effect of unilateral tubal block diagnosed by hysterosalpingogram on clinical pregnancy rate in intrauterine insemination cycles: systematic review and meta-analysis. BJOG. 2019 Jan;126(2):227-235. [↑](#footnote-ref-9)
9. Kim KH, Lee JY, Kang DH, Lee H, Seo JT, Cho KS. Impact of surgical varicocele repair on pregnancy rate in subfertile men with clinical varicocele and impaired semen quality: a meta-analysis of randomized clinical trials. Korean J Urol. 2013 Oct;54(10):703-9. [↑](#footnote-ref-10)
10. Kirby EW, Wiener LE, Rajanahally S, Crowell K, Coward RM. Undergoing varicocele repair before assisted reproduction improves pregnancy rate and live birth rate in azoospermic and oligospermic men with a varicocele: a systematic review and meta-analysis. Fertil Steril. 2016 Nov;106(6):1338-1343. [↑](#footnote-ref-11)
11. Esteves SC, Miyaoka R, Roque M, Agarwal A. Outcome of varicocele repair in men with nonobstructive azoospermia: systematic review and meta-analysis. Asian J Androl. 2016 Mar-Apr;18(2):246-53. [↑](#footnote-ref-12)
12. Sharpe A, Morley LC, Tang T, Norman RJ, Balen AH. Metformin for ovulation induction (excluding gonadotrophins) in women with polycystic ovary syndrome. Cochrane Database Syst Rev. 2019 Dec 17;12(12):CD013505. [↑](#footnote-ref-13)
13. Bordewijk EM, Ng KYB, Rakic L, Mol BWJ, Brown J, Crawford TJ, van Wely M. Laparoscopic ovarian drilling for ovulation induction in women with anovulatory polycystic ovary syndrome. Cochrane Database Syst Rev. 2020 Feb 11;2(2):CD001122. [↑](#footnote-ref-14)
14. Rao M, Zeng Z, Tang L. Maternal physical activity before IVF/ICSI cycles improves clinical pregnancy rate and live birth rate: a systematic review and meta-analysis. Reprod Biol Endocrinol. 2018 Feb 7;16(1):11. [↑](#footnote-ref-15)
15. Lensen SF, Wilkinson J, Leijdekkers JA, La Marca A, Mol BWJ, Marjoribanks J, Torrance H, Broekmans FJ. Individualised gonadotropin dose selection using markers of ovarian reserve for women undergoing in vitro fertilisation plus intracytoplasmic sperm injection (IVF/ICSI). Cochrane Database Syst Rev. 2018 Feb 1;2(2):CD012693. [↑](#footnote-ref-16)
16. Liu Y, Hu L, Fan L, Wang F. Efficacy of dehydroepiandrosterone (DHEA) supplementation for in vitro fertilization and embryo transfer cycles: a systematic review and meta-analysis. Gynecol Endocrinol. 2018 Mar;34(3):178-183. [↑](#footnote-ref-17)
17. Laganà AS, Vitagliano A, Noventa M, Ambrosini G, D'Anna R. Myo-inositol supplementation reduces the amount of gonadotropins and length of ovarian stimulation in women undergoing IVF: a systematic review and meta-analysis of randomized controlled trials. Arch Gynecol Obstet. 2018 Oct;298(4):675-684. [↑](#footnote-ref-18)
18. Zheng X, Lin D, Zhang Y, Lin Y, Song J, Li S, Sun Y. Inositol supplement improves clinical pregnancy rate in infertile women undergoing ovulation induction for ICSI or IVF-ET. Medicine (Baltimore). 2017 Dec;96(49):e8842. [↑](#footnote-ref-19)
19. Mizrachi Y, Horowitz E, Farhi J, Raziel A, Weissman A. Ovarian stimulation for freeze-all IVF cycles: a systematic review. Hum Reprod Update. 2020 Jan 1;26(1):118-135. [↑](#footnote-ref-20)
20. Tang H, Mourad S, Zhai SD, Hart RJ. Dopamine agonists for preventing ovarian hyperstimulation syndrome. Cochrane Database Syst Rev. 2016 Nov 30;11(11):CD008605. doi: 10.1002/14651858.CD008605. [↑](#footnote-ref-21)
21. Georgiou EX, Melo P, Brown J, Granne IE. Follicular flushing during oocyte retrieval in assisted reproductive techniques. Cochrane Database Syst Rev. 2018 Apr 26;4(4):CD004634. doi: 10.1002/14651858.CD004634. [↑](#footnote-ref-22)
22. Zeng M, Su S, Li L. The effect of laser-assisted hatching on pregnancy outcomes of cryopreserved-thawed embryo transfer: a meta-analysis of randomized controlled trials. Lasers Med Sci. 2018 Apr;33(3):655-666. [↑](#footnote-ref-23)
23. ESHRE Special Interest Group of Embryology and Alpha Scientists in Reproductive Medicine. The Vienna consensus: report of an expert meeting on the development of ART laboratory performance indicators. Reprod Biomed Online. 2017 Nov;35(5):494-510. [↑](#footnote-ref-24)
24. Busnelli A, Dallagiovanna C, Reschini M, Paffoni A, Fedele L, Somigliana E. Risk factors for monozygotic twinning after in vitro fertilization: a systematicreview and meta-analysis. Fertil Steril. 2019 Feb;111(2):302-317. [↑](#footnote-ref-25)
25. Hviid KVR, Malchau SS, Pinborg A, Nielsen HS. Determinants of monozygotic twinning in ART: a systematic review and a meta-analysis. Hum Reprod Update. 2018 Jul 1;24(4):468-483. [↑](#footnote-ref-26)
26. Vermey BG, Buchanan A, Chambers GM, Kolibianakis EM, Bosdou J, Chapman MG, Venetis CA. Are singleton pregnancies after assisted reproduction technology (ART) associated with a higher risk of placental anomalies compared with non-ART singleton pregnancies? A systematic review and meta-analysis. BJOG. 2019 Jan;126(2):209-218. [↑](#footnote-ref-27)
27. Lee E, Illingworth P, Wilton L, Chambers GM. The clinical effectiveness of preimplantation genetic diagnosis for aneuploidy in all 24 chromosomes (PGD-A): systematic review. Hum Reprod. 2015 Feb;30(2):473-83. [↑](#footnote-ref-28)
28. Munné S, Kaplan B, Frattarelli JL, Child T, Nakhuda G, Shamma FN, Silverberg K, Kalista T, Handyside AH, Katz-Jaffe M, Wells D, Gordon T, Stock-Myer S, Willman S; STAR Study Group. Preimplantation genetic testing for aneuploidy versus morphology as selection criteria for single frozen-thawed embryo transfer in good-prognosis patients: a multicenter randomized clinical trial. Fertil Steril. 2019 Dec;112(6):1071-1079. [↑](#footnote-ref-29)
29. Cornelisse S, Zagers M, Kostova E, Fleischer K, van Wely M, Mastenbroek S. Preimplantation genetic testing for aneuploidies (abnormal number of chromosomes) in in vitro fertilisation. Cochrane Database Syst Rev. 2020 Sep 8;9:CD005291. [↑](#footnote-ref-30)
30. Watters M, Noble M, Child T, Nelson S. Short versus extended progesterone supplementation for luteal phase support in fresh IVF cycles: a systematic review and meta-analysis. Reprod Biomed Online. 2020 Jan;40(1):143-150. [↑](#footnote-ref-31)
31. Mascarenhas M, Sunkara SK, Antonisamy B, Kamath MS. Higher risk of preterm birth and low birth weight following oocyte donation: A systematic review and meta-analysis. Eur J Obstet Gynecol Reprod Biol. 2017 Nov;218:60-67. [↑](#footnote-ref-32)
32. Jeve YB, Potdar N, Opoku A, Khare M. Donor oocyte conception and pregnancy complications: a systematic review and meta-analysis. BJOG. 2016 Aug;123(9):1471-80. [↑](#footnote-ref-33)
33. Masoudian P, Nasr A, de Nanassy J, Fung-Kee-Fung K, Bainbridge SA, El Demellawy D. Oocyte donation pregnancies and the risk of preeclampsia or gestational hypertension: a systematic review and metaanalysis. Am J Obstet Gynecol. 2016 Mar;214(3):328-39 [↑](#footnote-ref-34)
34. Wang Y, Anazodo A, Logan S. Systematic review of fertility preservation patient decision aids for cancer patients. Psychooncology. 2019 Mar;28(3):459-467. [↑](#footnote-ref-35)
35. Cortessis VK, Azadian M, Buxbaum J, Sanogo F, Song AY, Sriprasert I, Wei PC, Yu J, Chung K, Siegmund KD. Comprehensive meta-analysis reveals association between multiple imprinting disorders and conception by assisted reproductive technology. J Assist Reprod Genet. 2018 Jun;35(6):943-952. [↑](#footnote-ref-36)
36. Chen L, Yang T, Zheng Z, Yu H, Wang H, Qin J. Birth prevalence of congenital malformations in singleton pregnancies resulting from in vitro fertilization/intracytoplasmic sperm injection worldwide: a systematic review and meta-analysis. Arch Gynecol Obstet. 2018 May;297(5):1115-1130. [↑](#footnote-ref-37)
37. Liang Y, Chen L, Yu H, Wang H, Li Q, Yu R, Qin J. Which type of congenital malformations is significantly increased in singleton pregnancies following after *in vitro* fertilization/intracytoplasmic sperm injection: a systematic review and meta-analysis. Oncotarget. 2017 Dec 25;9(3):4267-4278. [↑](#footnote-ref-38)
38. Hoorsan H, Mirmiran P, Chaichian S, Moradi Y, Hoorsan R, Jesmi F. Congenital Malformations in Infants of Mothers Undergoing Assisted Reproductive Technologies: A Systematic Review and Meta-analysis Study. J Prev Med Public Health. 2017 Nov;50(6):347-360. [↑](#footnote-ref-39)
39. Giorgione V, Parazzini F, Fesslova V, Cipriani S, Candiani M, Inversetti A, Sigismondi C, Tiberio F, Cavoretto P. Congenital heart defects in IVF/ICSI pregnancy: systematic review and meta-analysis. Ultrasound Obstet Gynecol. 2018 Jan;51(1):33-42. [↑](#footnote-ref-40)
